# Identification of key pathways and genes in carotid atherosclerosis through bioinformatics analysis of RNA-seq data

**DOI:** 10.18632/aging.202943

**Published:** 2021-05-11

**Authors:** Zhongchen Li, Jiheng Hao, Kun Chen, Qunlong Jiang, Peijian Wang, Xiaohui Xing, Jiyue Wang, Yinjiang Zhang, Yilei Xiao, Liyong Zhang

**Affiliations:** 1 Department of Neurosurgery, Liaocheng People’s Hospital, Shandong Provincial Hospital, Cheeloo College of Medicine, Shandong University, Liaocheng 252000, Shandong Province, P.R. China; 2 School of Pharmacy, Minzu University of China, Zhongguancun, Beijing 100081, P.R. China

**Keywords:** carotid atherosclerosis, RNA-seq, enrichment analysis, hub gene, bioinformatics analysis

## Abstract

While acknowledging carotid atherosclerosis (CAS) as a risk factor for ischemic stroke, reports on its pathogenesis are scarce. This study aimed to explore the potential mechanism of CAS through RNA-seq data analysis. Carotid intima tissue samples from CAS patients and healthy subjects were subjected to RNA-seq analysis, which yielded, 1,427 differentially expressed genes (DEGs) related to CAS. Further, enrichment analysis (Gene Ontology, KEGG pathway, and MOCDE analysis) was performed on the DEGs. Hub genes identified via the protein-protein interaction network (PPI) were then analyzed using TRRUST, DisGeNET, PaGenBase, and CMAP databases. Results implicated inflammation and immunity in the pathogenesis of CAS. Also, lung disease was associated with CAS. Hub genes were expressed in multiple diseases, mainly regulated by RELA and NFKB1. Moreover, three small-molecule compounds were found via the CMAP database for management of CAS; hub genes served as potential targets. Collectively, inflammation and immunity are the potential pathological mechanisms of CAS. This study implicates CeForanide, Chenodeoxycholic acid, and 0317956-0000 as potential drug candidates for CAS treatment.

## INTRODUCTION

Carotid atherosclerosis (CAS) is the most prevalent type of atherosclerosis (AS), at the bifurcation point of the external carotid artery (ECA) and internal carotid artery (ICA) [1]. It is characterized by artery stenosis attributed to the deposition of atherosclerotic plaque comprising a cholesterol layer on the carotid artery wall [2]. Cerebrovascular disease is described as a group of conditions including ischemic stroke, aneurysm, and vascular malformation. Ischemic stroke is the most predominant form of cerebrovascular disease [3], mainly caused by the rupture of CAS plaque. The rising incidence of CAS over years warrants further exploration of its pathogenesis for stroke prevention [4, 5]. Numerous studies found that dyslipidemia, vascular endothelial cell dysfunction, lipid accumulation, excessive oxidative stress reaction, and inflammatory reaction are associated with CAS [6–10]. However, the molecular mechanism of CAS pathogenesis is elusive, which warrants further exploration. Despite the clinical prescription of statins to alleviate CAS through blood lipid regulation, they induce myalgia and rhabdomyolysis caused by high creatine kinase levels [11]. Clinicians recommend surgical treatment, including percutaneous balloon dilatation, stenting, bypass grafting, etc., for patients with severe lesions. However, medication still is required post-surgery. Prognosis is bleak for patients whose arteries of vital organs are involved [12].

Transcriptomic sequencing technology (RNA-seq) is adopted to evaluate the binding of proteins to RNA in the natural state of cells. RNA-seq enables researchers to explore the function and structure of genes from the genomic level, to elucidate the molecular mechanism of specific biological processes and the occurrence of diseases. This technique has special advantages in the analysis of gene fusion, splicing variation, and gene expression spectrum [13, 14] and is a powerful technology for studying pathogenesis in clinical diagnosis and pharmacological research. Previously, for instance, by Pan et al., RNA-seq technology was applied to explore hub genes and pathways in atherosclerosis model of Tibetan miniature pigs fed with a high-fat diet [15]. Elsewhere, Cochran et al. adopted single-cell RNA-seq technology to explore transcriptional landscape and heterogeneity of aortic macrophages in mouse atherosclerosis [16].

**Figure 1:**
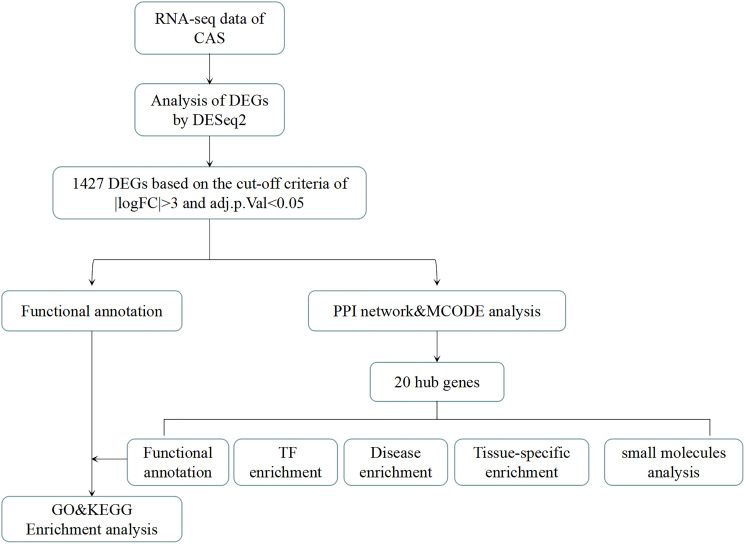
**The analysis flow chart of the study.** CAS: Carotid atherosclerosis. DEGs: differentially expressed genes. DEseq2: R package for transcriptional sequencing. FC: Fold change. |logFC|>3: The logarithm of the ratio of mRNA expression in CAS patients to that of healthy people is more than 3 times. Adj.*p*.Val: corrected *p*-value. PPI: protein-protein interaction. TF enrichment: transcription factor enrichment.

Herein, to explore the possible mechanism of CAS, we integrated RNA-seq technology with bioinformatics analysis to elucidate differences in gene expression profile between CAS patients and healthy subjects. As shown in Figure 1, related sequencing data were obtained through RNA-seq analysis. Using the R package, differentially expressed genes (DEGs) between CAS experimental group and corresponding healthy controls were identified. Next, we performed functional cluster analysis on DEGs, and the construction of a PPI network to identify hub genes. We further predicted small molecule drug compounds that potentially interact with hub genes using the CMAP online database. Findings from this study will enrich our understanding of the underlying molecular pathologic mechanisms of CAS.

## RESULTS

### Basic analysis of sequencing data

After filtering the original data, checking the sequencing error rate, and verifying the distribution of GC content, the clean reads for follow-up analysis were obtained. Carotid atherosclerosis samples ASA_1 and ASA_2 generated 57.14, and 72.76 million clean reads, respectively. Normal control samples NA_1, NA_2, and NA_3 generated 59.84, 70.13, and 59.98 million clean reads, respectively (Table 1). Quality-controlled clean reads were compared to the human reference genome. Results of the comparison of each sample in this project with the human reference genome are presented in Table 2. Total mapped reads for ASA_1 and ASA_2 were 96.08 and 94.70%, whereas those for NA_1, NA_2, and NA_3 were 94.95, 96.28, and 95.99%, respectively.

**Table 1:** The summary of sequencing data quality.

**sample**	**library**	**raw_reads**	**clean_reads**	**clean_bases**	**error_rate**	**Q20**	**Q30**	**GC_pct**
ASA_1	FRAS190011021-2a	58340980	57142184	8.57G	0.03	97.23	92.51	52
ASA_2	FRAS190023363-1a	73973466	72756502	10.91G	0.03	96.65	90.57	51.44
NA_1	FRAS190011024-2a	60927190	59837526	8.98G	0.03	96.39	90.05	51.68
NA_2	FRAS190023357-1a	71565888	70129076	10.52G	0.03	97.12	92.25	51.58
NA_3	FRAS190023358-1a	61095962	59977760	9.0G	0.03	96.93	91.93	51.6

Library: Library number. Raw_reads: The number of reads in the original data. Clean_reads: The number of filtered reads from the original data. Clean_bases: The number of bases filtered from the original data. Error_rate: Overall sequencing error rate of the data. Q20: The percentage of bases with a Phred value greater than 20 in the total. Q30: The percentage of bases with a Phred value greater than 30. GC_pct: The percentage of G and C in clean reads. ASA: Carotid atherosclerosis group. NA: Normal control group.

**Table 2:** The statistics of sample contrast ratio.

**sample**	**total_reads**	**total_map**	**unique_map**	**multi_map**	**read1_map**	**read2_map**	**positive_map**	**negative_map**	**splice_map**	**unsplice_map**	**proper_map**
ASA_1	57142184	54902494 (96.08%)	53646761 (93.88%)	1255733 (2.2%)	26926425 (47.12%)	26720336 (46.76%)	26813961 (46.92%)	26832800 (46.96%)	22789408 (39.88%)	30857353 (54.0%)	52002374 (91.01%)
ASA_2	72756502	68901477 (94.7%)	67258317 (92.44%)	1643160 (2.26%)	33786473 (46.44%)	33471844 (46.01%)	33603434 (46.19%)	33654883 (46.26%)	29413322 (40.43%)	37844995 (52.02%)	64681242 (88.9%)
NA_1	59837526	56817623 (94.95%)	55508995 (92.77%)	1308628 (2.19%)	28024601 (46.83%)	27484394 (45.93%)	27717719 (46.32%)	27791276 (46.44%)	22786703 (38.08%)	32722292 (54.69%)	53405306 (89.25%)
NA_2	70129076	67521537 (96.28%)	65978771 (94.08%)	1542766 (2.2%)	33167591 (47.3%)	32811180 (46.79%)	32952645 (46.99%)	33026126 (47.09%)	27774208 (39.6%)	38204563 (54.48%)	63953182 (91.19%)
NA_3	59977760	57573643 (95.99%)	56339554 (93.93%)	1234089 (2.06%)	28325924 (47.23%)	28013630 (46.71%)	28139754 (46.92%)	28199800 (47.02%)	23053533 (38.44%)	33286021 (55.5%)	54544526 (90.94%)

Total_reads: The number of clean reads of sequencing data after quality control. Total map: The number and percentage of reads that have been aligned to the genome. Unique_map: The number and percentage of reads that were aligned to the unique location of the reference genome. Multi_map: The number and percentage of reads that are aligned to multiple locations in the reference genome. Read1_map: The number and percentage of read1 matched to the reference genome. Read2_map: The number of read2 matched to the reference genome and its percentage. Positive_map: The number and percentage of reads that were aligned to the positive strand of the reference genome. Negative_map: The number and percentage of reads aligned to the negative strand of the reference genome. Splice_map: The number of reads and their percentages that were extracted and aligned to the genome. Unsplice_map: Number of reads and their percentages that have not been parsed onto the genome. Proper_map: The number and percentage of reads paired read1 and read1 that are simultaneously aligned to the genome. ASA: Carotid atherosclerosis group, NA: Normal control group.

### Gene expression analysis

**Figure 2:**
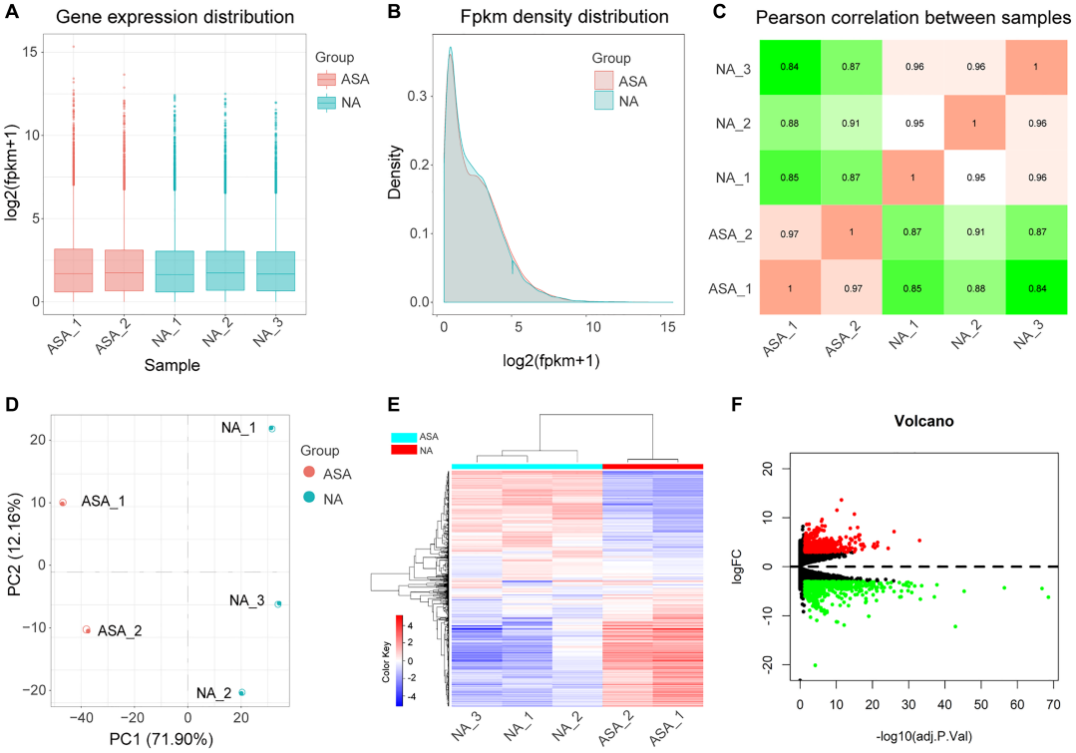
**Analysis of DEGs.** Boxplot (**A**) and density plot (**B**) showing that gene expression levels of different samples are evenly distributed. (**C**) Correlation diagram of gene expression level between samples indicates that the correlation coefficient between samples of the same group is high and the sample selection is reliable. (**D**) Principal component analysis diagram (PCA) showing that samples of different groups were significantly different, and samples within the same group were relatively uniform. (**E**) Heat map of DEGs, red represents high expression, blue represents low expression. (**F**) Volcano map of DEGs, screening parameters were |logFC|>3, adj.*P*.Val <0.05, 710 up-regulated genes (red dots), and 717 down-regulated genes (green dots) were identified, genes with no significant changes were labeled as black dots. ASA: Carotid atherosclerosis group. NA: Normal control group. FC: Fold change. Adj.*p*.Val: corrected *p*-value.

Box and density diagrams depicted uniform distribution of gene expression levels in different samples (Figure 2A–2B). Based on correlation analysis of gene expression levels among samples, the experiment was reliable with accurate sample selection (Figure 2C). Principal component analysis (PCA) revealed that samples were scattered among groups and clustered within groups (Figure 2D).

### Identification of differentially expressed genes

After normalizing the sequencing data, we constructed the heat map (Figure 2E). A total of 1427 DEGs were identified after screening at |logFC|>3 and adj.*p*.Val <0.05 (Figure 2F, Supplementary Table 1); 710 DEGs and, 717 DEGs were up-regulated and down-regulated, respectively.

### GO and KEGG enrichment analysis of DEGs

**Figure 3:**
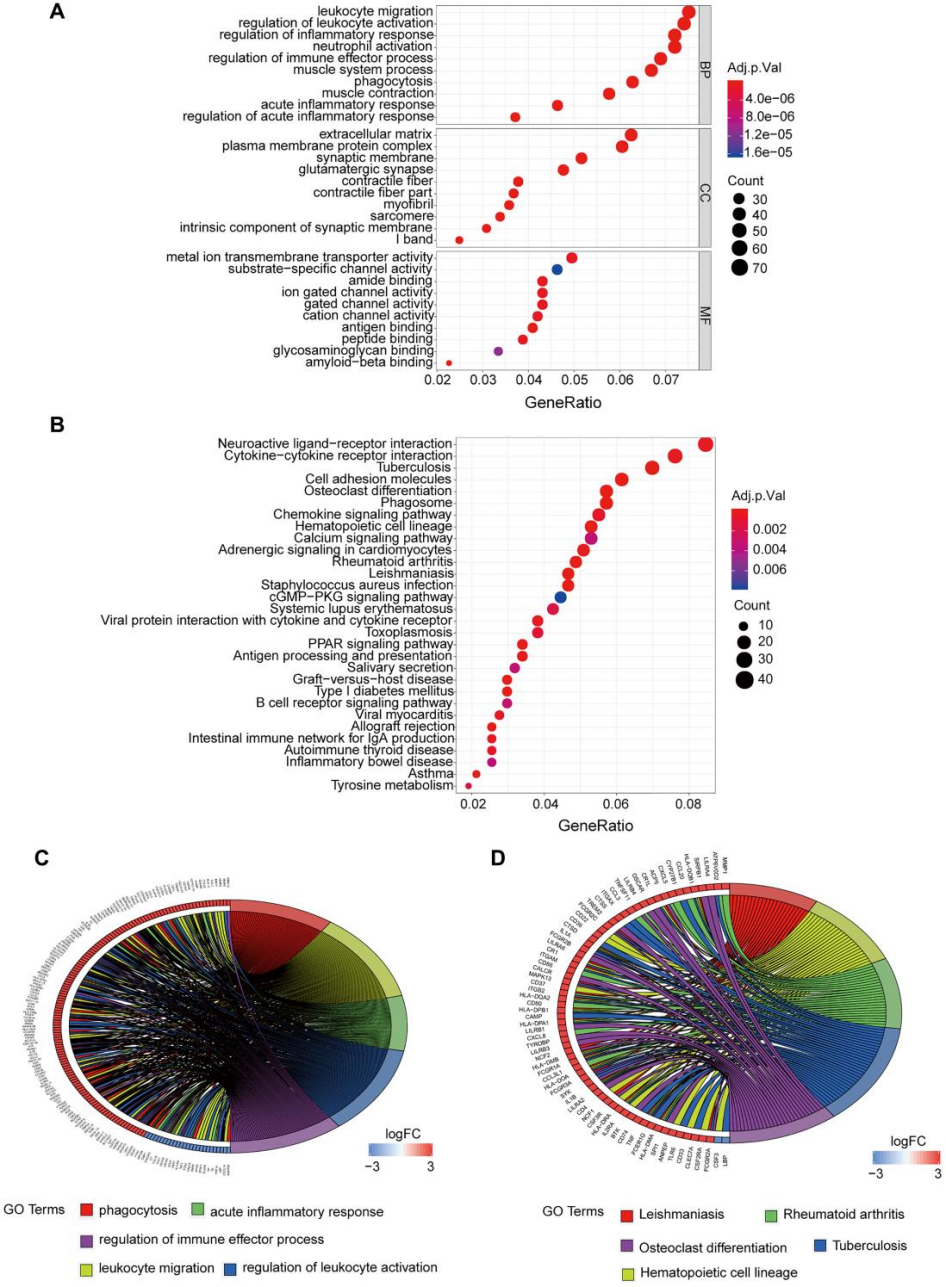
**GO and KEGG enrichment analysis of DEGs.** (**A**) GO enrichment analysis of DEGs, BP represents the biological process, CC represents the cellular component, and MF represents the molecular function. (**B**) KEGG pathway analysis of DEGs, adj.*p*.Val is corrected *p*-value, the count represents the number of DEGs. (**C**) Interaction between DEGs and top 5 terms of GO entries. (**D**) Interaction between DEGs and top 5 disease pathways in KEGG. DEGs: Differentially expressed genes. KEGG: Kyoto Encyclopedia of Genes and Genomes. FC: Fold change. Adj.*p*.Val: corrected *p*-value.

GO analysis revealed that biological processes (BP) of these targets were mainly enriched in leukocyte migration, regulation of activation, and inflammatory response. Enriched cell components (CC) included extracellular matrix, plasma membrane protein complex, and synaptic membrane. Molecular functions (MF) were mainly enriched in Metal ion Transmembrane Transporter activity, Substrate-specific channel activity, and amide binding (Figure 3A). KEGG pathway analysis demonstrated that enriched pathways for these targets are implicated in neuroactive ligand-receptor interaction, cytokine-cytokine receptor interaction, and cell inflammatory molecules (Figure 3B).

Cluster analysis for the top five GO terms explored the associations of genes such as MMP12, MMP8, and MMP9 with phagocytosis, leukocyte migration, acute response, regulation of immune activation, and regulation of immune effector process (Figure 3C). We performed cluster analysis for the top 5 KEGG pathways to explore the association of genes such as LBP, CSF3, and FCGR2A with leishmaniasis, hematopoietic cell, strain, tuberculosis, and osteoclast differentiation (Figure 3D).

### Enrichment analysis of DEGs in MCODE

**Figure 4:**
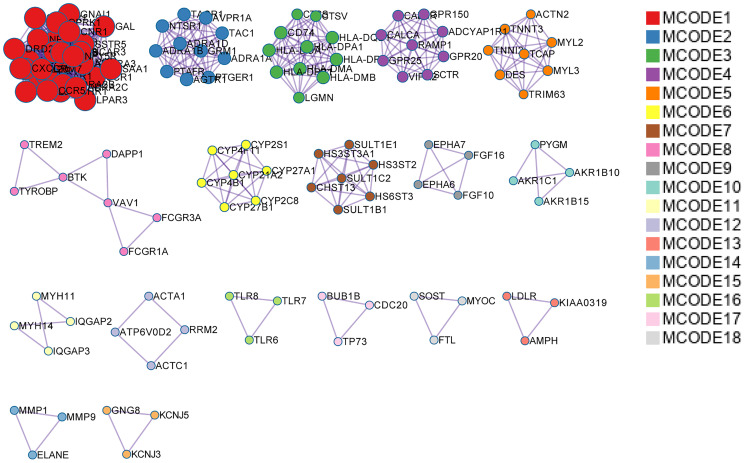
**MCODE analysis of DEGs.** DEGs were divided into 18 modules based on functions, and protein-protein interaction networks were constructed for DEGs of each module. DEGs: Differentially expressed genes.

MCODE analysis demonstrated that DEGs were grouped into 18 modules. Briefly, Module 1 was comprised of 29 genes, including GAL, GNAl1, and OPRK1, etc. Module 2 comprised 11 genes, including TACR1, NTSR1, and TAC1, etc. Module 3 comprised 11 genes including CD74, CTSS, and CTSV, etc. (Figure 4). We did KEGG enrichment analysis on the 18 modules. Pathways in module 1 were mainly associated with neuroactive ligand-receptor interaction, whereas those in module 2 and module 3 were mainly enriched in cAMP signaling and chemokine signaling, respectively, and so on (Table 3).

**Table 3:** The results of MCODE enrichment analysis.

**MCODE**	**GO**	**Description**	**Log10 (*P*)**
MCODE_1	hsa04080	Neuroactive ligand-receptor interaction	−21.5
MCODE_1	hsa04024	cAMP signaling pathway	−12
MCODE_1	hsa04062	Chemokine signaling pathway	−10.5
MCODE_2	hsa04020	Calcium signaling pathway	−20.3
MCODE_2	hsa04080	Neuroactive ligand-receptor interaction	−18.5
MCODE_2	hsa04270	Vascular smooth muscle contraction	−8.9
MCODE_3	hsa04612	Antigen processing and presentation	−24.2
MCODE_3	hsa05310	Asthma	−18.1
MCODE_3	hsa05152	Tuberculosis	−17.6
MCODE_4	hsa04080	Neuroactive ligand-receptor interaction	−5.7
MCODE_5	hsa05410	Hypertrophic cardiomyopathy (HCM)	−5.7
MCODE_5	hsa05414	Dilated cardiomyopathy	−5.6
MCODE_7	hsa00534	Glycosaminoglycan biosynthesis - heparan sulfate / heparin	−7.5
MCODE_8	hsa04380	Osteoclast differentiation	−10.1
MCODE_8	hsa04662	B cell receptor signaling pathway	−6.1
MCODE_8	hsa04666	Fc gamma R-mediated phagocytosis	−5.8
MCODE_11	hsa04810	Regulation of actin cytoskeleton	−5.6
MCODE_15	hsa05032	Morphine addiction	−7.3
MCODE_15	hsa04713	Circadian entrainment	−7.2
MCODE_15	hsa04723	Retrograde endocannabinoid signaling	−7.2
MCODE_16	hsa04620	Toll-like receptor signaling pathway	−7.1

### Constructing PPI network and screening for hub genes

**Figure 5:**
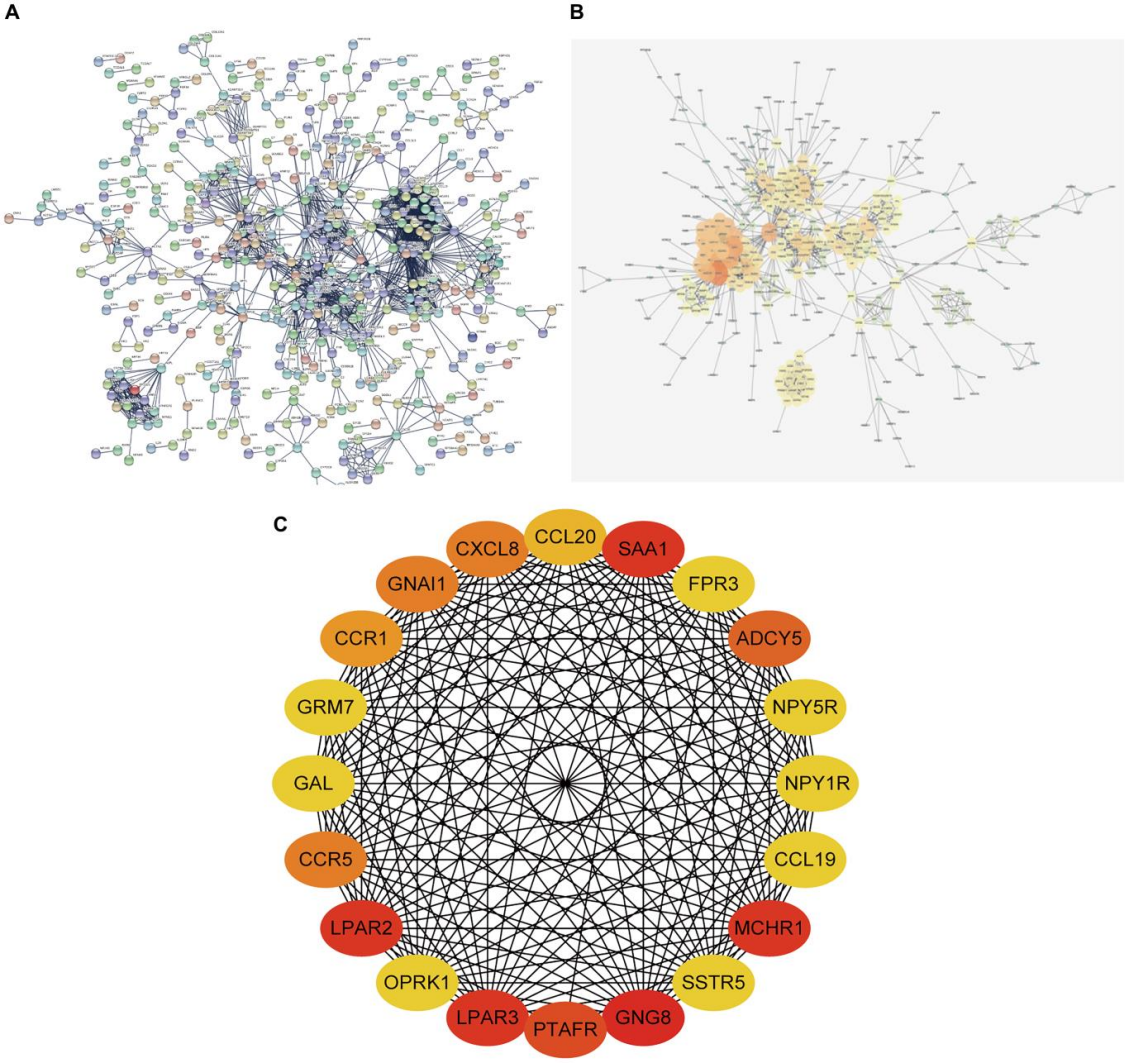
**PPI network constructing and hub gene screening of DEGs.** (**A**) PPI network constructed using STRING Online Database contained 1055 nodes and 1883 edges. (**B**) PPI network was optimized through Cytoscape software. (**C**) 20 hub genes identified through degree method in cytoHubba. PPI network: protein-protein interaction network.

The STRING online Database was employed to construct the PPI network of DEGs (Figure 5A), which was then visualized and optimized using Cytoscape software (Figure 5B). Analysis of hub genes was achieved using the degree method in cytoHubba; the top 20 genes were identified as hub genes (Figure 5C).

### Bioinformatics analysis of hub genes

**Figure 6:**
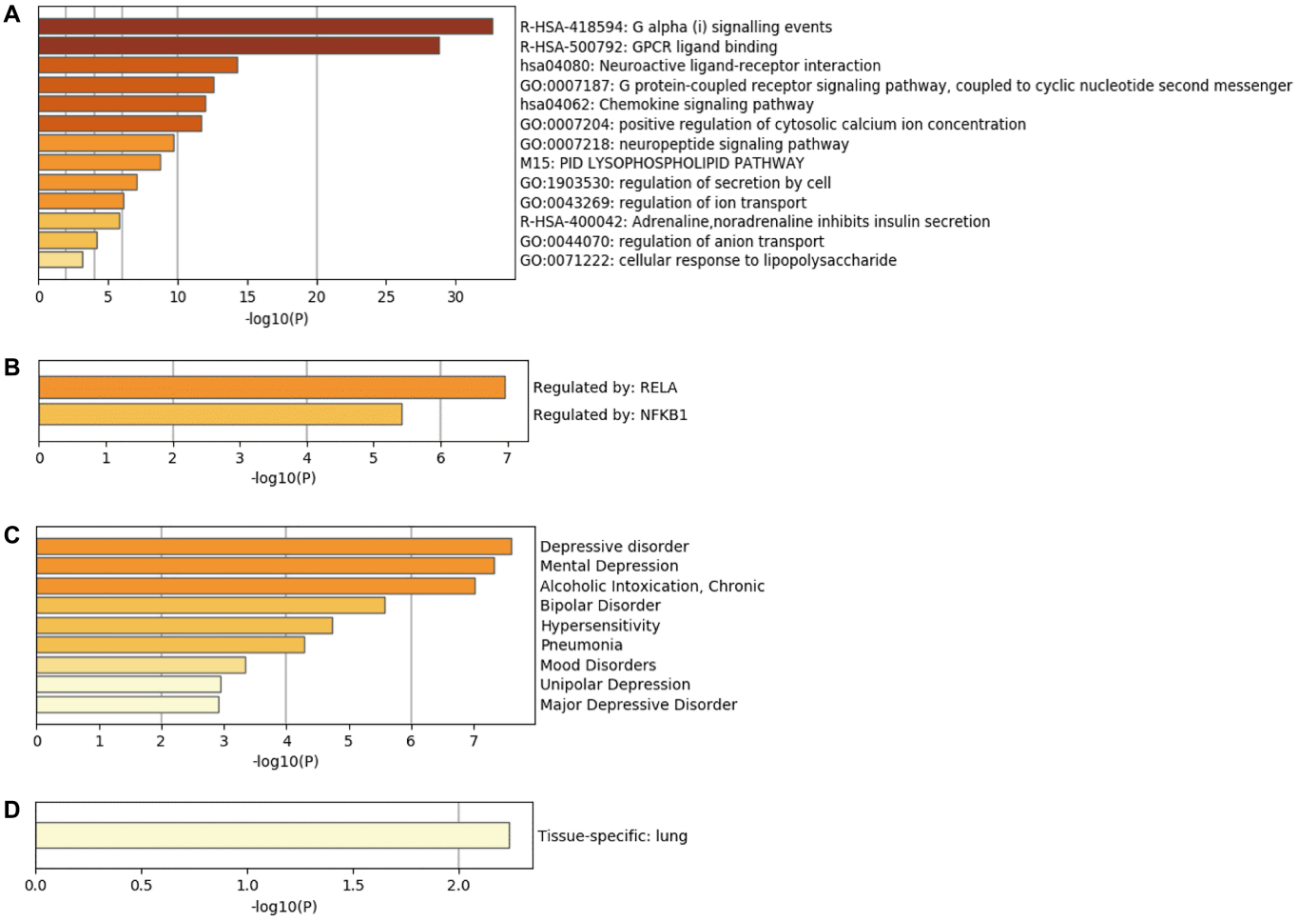
**Bioinformatics analysis of hub genes.** (**A**) Biological functions of hub genes analyzed through Metascape database. (**B**) Enrichment of transcriptional regulators of hub genes using the TRRUST database. (**C**) DisGeNET database enrichment analysis of diseases involving hub genes. (**D**) Tissue characteristics of hub genes as analyzed using PaGenBase database.

Enrichment analysis of top 20 hub genes in the Metascape database revealed that these genes are mainly associated with Galpha (i) signaling events, GPCR ligand binding, and neuroactive ligand-receptor interaction (Figure 6A). TRRUST database analysis uncovered RELA and NF-κB1 to be the main transcription factors regulating the hub genes (Figure 6B). DisGeNET database disease enrichment analysis demonstrated that the hub genes were associated with depressive disorder, mental depression, and alcoholic intoxication (Figure 6C). Besides, PaGenBase database tissue characteristic enrichment analysis showed that hub genes were mainly enriched in the lung (Figure 6D). Analysis of the CMAP database showed that CeForanide, Chenodeoxycholic acid, and 0317956-0000 compounds (Table 4) potentially exerted a negative regulatory effect on hub genes. We deduced that these three drugs are potential candidates for the management of carotid atherosclerosis.

**Table 4:** The result of CMAP database analysis.

**rank**	**cmap name**	**mean**	***n***	**enrichment**	***p***	**specificity**	**percent non-null**
1	corticosterone	0.627	4	0.861	0.0005	0	100
2	ceforanide	−0.606	4	−0.831	0.00147	0.006	100
3	chenodeoxycholic acid	−0.491	4	−0.83	0.00155	0.0308	100
4	mebendazole	0.536	5	0.736	0.00296	0.0766	80
5	cotinine	0.4	6	0.669	0.00364	0	83
6	Prestwick-984	0.62	4	0.789	0.00392	0.0227	100
7	5230742	0.709	2	0.948	0.00489	0.0078	100
8	prenylamine	0.646	4	0.773	0.00501	0.0739	100
9	0317956-0000	−0.454	8	−0.573	0.00501	0.0565	62
10	flunisolide	0.51	6	0.646	0.00572	0	83
11	sodium phenylbutyrate	−0.342	7	−0.604	0.00587	0.0196	71
12	levodopa	0.502	5	0.691	0.00711	0.0088	80
13	mepenzolate bromide	0.37	5	0.686	0.00771	0.0199	60
14	hesperidin	0.373	4	0.744	0.0081	0.0073	75

## DISCUSSION

Carotid atherosclerosis is a vascular disease ascribed to lipid deposition. It is characterized by three pathological and anatomic stages, including the initial stage, progressive stage, and terminal stage [17]. CAS plaque rupture or erosion is a high-risk factor for cardiovascular disease and accounts for most deaths in CAS patients [18, 19]. Transcriptomic sequencing (RNA-seq) is a highly useful tool for studying gene expression. The overall transcription level of genes differentially expressed genes and variable splicing events can be explored via mRNA sequence analysis of cells. Further, targeted genes and mediated signaling pathways can be revealed via RNAseq. In recent years, RNA-seq, a high-throughput sequencing method has been applied widely to evaluate the pathogenesis of numerous diseases. In particular, Liu et al. evaluated the preventive effect of honeysuckle on acute lung injury caused by lipopolysaccharide using RNA-seq [20], whereas Esh et al. used RNA-seq proteomics and global post-translational modification (G-PTM) search strategy to explore the prognosis of esophageal cancer at the molecular level. Esh and colleagues found proteome variation of esophageal squamous cell carcinoma, and protein modification could function as a potential biomarker [21]. Currently, there are still limited reports on the application of RNA-seq technology in CAS.

In the present study, we employed the R package (DESeq2) to analyze the sequencing data set and yielded 1,427 DEGs (710 up-regulated and 717 down-regulated). This finding demonstrates that carotid atherosclerosis is associated with multiple gene regulatory changes. Of note, selected DEGs have been implicated in various diseases. Tacke F et al. found that expression of CCR5 was upregulated. They tracked chemokine receptors using monocyte subpopulation derived from AS plaque in APOE^-^ mice [22]. mRNA expression levels of NPY and NPY5R were upregulated in the hippocampal dentate gyrus of type 1 diabetic rats. However, administration with Sitagliptin relieved the mild changes in the hippocampal NPY system of early type 1 diabetic rats [23]. We can, therefore, attest that DEGs identified in the present study are potential biomarkers and therapeutic targets for CAS.

Furthermore, GO and KEGG enrichment analysis revealed that expression changes of these DEGs influenced signal transduction pathways associated with inflammation and immune response, for example, cytokine-mediated signal transduction and inflammatory response. Previous studies found close associations between inflammation, CAS, and plaque instability [24]. Inflammation-induced by cholesterol crystals (CC) is the crucial step in the progression of CAS. CCs mediate complement activation and induce plaque inflammation by activating NACHT, LRR, and PYD domains harboring protein 3 (NLRP3) [25]. Elsewhere, Gregersen reported that plasma IL-27 levels in CAS patients were significantly higher than that of normal subjects. Moreover, the expression of IL-27 and IL-27 receptor genes in plaques was significantly high. Previous *in vitro* studies show that IL-27 promotes activation of NLRP3 inflammasomes in monocytes [26]. These findings suggest that inflammation is critical in CAS development. Therefore, our selected DEGs are potential markers for exploring the pathological mechanism of CAS vasculitis.

Function-based grouping of the DEGs into 18 modules affirmed their different effects in the pathological process of CAS. Also, the regulation of module genes is interrelated. In a nutshell, the pathological mechanism of CAS is a complex process, associated with ligand-receptor interaction of the nervous system, cAMP signaling pathways, chemokine signaling pathways, and other biological processes. From the constructed PPI network of the DEGs, we identified 20 hub genes. Previous reports show that some of these hub genes are associated with AS. A previous analysis of mRNA and protein levels of GAL-3 in carotid endarterectomy specimens from CAS patients showed that expression levels were significantly higher compared to those in stable plaque areas. Similar findings were reported using animal cell experiments [27]. Qin reported that G31P, an antagonist of the cxcl-8 receptor, inhibited dyslipidemia in AS mice and delayed the development of AS [28].

Multiple enrichment analysis of hub genes using four databases revealed that these genes were associated with several biological processes and pathogenesis of various diseases. These findings implicate hub genes as key regulatory players in the human body. Further analysis using the Metascape database demonstrated that hub genes are associated with numerous biological functions, such as neuroligin-receptor interactions, as was reported previously [23]. NFKB1 (NF-κB) was found to be the primary regulatory transcription factor of hub genes based on the results of TRRUST database analysis. A previous experiment showed that inhibition of NF-κB phosphorylation could reduce the expression of related chemokines, reduce inflammatory response significantly, and impede the malignant development of AS [29]. Additionally, DisGeNET database analysis implicated hub genes in the development of depression and other diseases. Kobayashi reported that G protein signal transduction regulator 8 (RGS8) participated in the regulation of depressor-like behaviors by tuning ciliate MCHR1 expressed in the hippocampus CA1 region [30]. PaGenBase database analysis demonstrated that hub genes were mainly enriched in the lung, suggesting that the development of AS is potentially correlated with lung function. Liu found that allergic pulmonary inflammation promoted AS lesions in apoE-deficient mice [31]. The multi-angle enrichment analysis helps in understanding the pathogenesis of CAS from multiple aspects.

Furthermore, using the CMAP database, we identified three small molecular compounds (CeForanide, Chenodeoxycholic acid, and 0317956-0000) that potentially interact with central genes. Reports from clinical trials show that ceforanide has a satisfactory antibacterial effect, and is effective in treating skin and soft tissue, pulmonary and urinary tract infections, bone and joint infections, and endocarditis [32]. The small molecule drugs that we screened may exert potential activity against atherosclerosis.

In conclusion, the present study adopted RNA-seq technology and bioinformatics analysis to explore the pathogenesis of CAS. It demonstrates that inflammation, immune response, and neuroligand-receptor interaction are associated with the development of CAS. Also, 20 hub genes and 3 candidate small-molecule drugs for CAS treatment have been uncovered. However, the presence of a female sample in the normal group used for sequencing may have impacted the results. Therefore further studies are warranted to explore the pathogenesis of CAS and the activity of selected drugs. These findings enrich our understanding of the pathogenesis of CAS and offer a basis for clinical diagnosis and treatment of CAS.

## MATERIALS AND METHODS

### Patients and samples

We analyzed two CAS vessels of CAS patients and 3 carotid artery vessels of healthy subjects. All samples were collected from subjects who visited Liaocheng People's Hospital between February 2018 and August 2018. Each subject gave consent to undergo an electrocardiogram (ecg), echocardiogram, head, and chest CT, CTA, and other examinations. In the experimental group, blood vessels were acquired from CAS patients CAS with stroke and presenting with dizziness, verbal slurred speech, limb dysfunction, and other manifestations of cerebral ischemia. After admission, patients underwent craniocerebral magnetic resonance imaging, head and neck CTA, and whole-brain angiography, which conformed to the latest guide on diagnosis and treatment of atherosclerosis. CAS plaques and intima of vessels were obtained from patients diagnosed with CAS, particularly those who accepted treatment via carotid endarterectomy. These patients were categorized in the observation group. In the control group, we obtained blood vessels from patients who succumbed to acute craniocerebral trauma but had been in good health after active treatment. Part of the artery of family members who volunteered to donate organs was retained as healthy control, though the organ was donated. Detailed clinical information of all participants is outlined in Table 5. Approval for this study was issued by the Ethics Committee of the hospital. All patients or their family members signed informed consent.

**Table 5:** Clinical information for all participants.

**index**	**gender**	**weight (kg)**	**height (cm)**	**age (years)**	**blood pressure (mmHg)**	**blood glucose (mmol/L)**
ASA_1	male	72	174	61	135/85	6.2
ASA_2	male	68	168	58	142/83	5.6
NA_1	male	66	163	56	128/78	5.2
NA_2	female	74	171	53	137/76	5.8
NA_3	male	77	177	55	122/81	4.9

Abbreviations: ASA: Carotid atherosclerosis group, NA: Normal control group.

### Tissue sample acquisition

The anterior edge of the sternocleidomastoid muscle was cut longitudinally under general anesthesia. The skin, subcutaneous, and platysma muscles were then cut layer by layer to expose and open the carotid sheath. The common carotid artery, external cervical artery, and superior thyroid artery were then blocked. Blood vessels were cut longitudinally or obliquely along the beginning of the internal carotid artery after blocking the internal carotid artery under the microscope. Intima and plaques were separated from the bifurcation to the distal end of the internal carotid artery with nerve stripping ions. Plaques of the common carotid artery and external carotid artery were stripped down in turn. The arterial lumen was washed with heparin saline repeatedly. The internal carotid artery was sutured continuously, then opened to exhaust thoroughly before knotting. After inhibiting gas occlusion, it was blocked. Blood flow of external carotid artery, superior thyroid artery, common carotid artery, and internal carotid artery were restored sequentially. We further verified that there is no blood osmosis in the anastomosis and then closed the incision layer by layer. After removing the intima and plaque of the carotid artery, plaque specimens were placed in an EP tube and frozen in liquid nitrogen. *In vitro* cryopreservation of specimen was conducted in 5 minutes.

### RNA extraction

Total RNA was extracted from samples using TRIzol reagent (Invitrogen, Carlsbad, CA, USA) following the manufacturer's protocol. RNA concentration and purity were assessed using a Nanodrop ND-2000 spectrophotometer (Thermo Fisher Scientific, Wilmington, DE, USA). RNA integrity was evaluated using 2% agarose gel. Agilent 2100 bioanalyzer (Agilent, Palo Alto, CA, USA) was used to obtain RNA integrity values (RIN).

### Transcriptome deep sequencing

After total RNA treatment with DNase I, mRNA was enriched with Oligo D (T) magnetic beads (New England Biolabs, Inc., Ipswich, MA, USA), and digested into short fragments. mRNA fragment was used as a template, whereas random oligonucleotide was used as a primer. The first strand of cDNA was synthesized using the M-MuLV reverse transcriptase system. Then, the RNA strand was degraded using RNaseH. The second strand was synthesized from dNTPs under the DNA polymerase I system. The double-stranded cDNA was purified, end-repaired, and “a-tailed” for adapter ligation. AMPure XP beads were used to screen out 250-300bp of cDNA for PCR amplification, followed by purification of the PCR products to obtain the library. Agilent 2100 Bioanalyzer (Agilent Technologies, Inc.) and ABI StepOnePlus Real-time PCR System (Applied Biosystems; Thermo Fisher Scientific, Inc.) were applied to quantify and identify the sample library for quality control. Sequencing of mRNA libraries was achieved with Illumina HiSeq TM 2000 (Illumina, Inc., San Diego, CA, USA).

### Data quality control

Sequences of the raw data containing a small number of reads with sequencing adaptors or low-quality sequences were filtered out. Since the sequencing process is prone to machine error, we established the quality of sequencing data based on sequencing error rate distribution examination. GC content distribution was also determined. HISAT2 [33] was employed to map clean reads to the reference genome (Ensemble GRCh38.p7).

### Differential gene screening

We evaluated the distribution of gene expression levels in different samples. A correlation diagram of gene expression levels between samples was applied to verify the reliability of the experiment and the accuracy of sample selection. Inter-group differences and intra-group sample duplications were evaluated via Principal component analysis (PCA). DESeq2 package in R software (R version 3.6.1: https://www.r-project.org/) was adopted to explore the differential gene expression [34]. Original read count was standardized (normalization), then the BH method was used for multiple hypotheses testing corrections. Differentially expressed genes (DEGs) were screened using the conditions, |logFC|>3, adj.*p*.Val <0.05. A heatmap of the selected DEGs was constructed using the pheatmap package [35].

### GO and KEGG pathway analysis of differential genes

Biological processes (BP), cellular component (CC), and molecular function (MF) categories were explored through GO analysis. KEGG (http://www.genome.jp/) analysis is a systematic approach to assess gene function for the discovery of bioregulatory pathways. Herein, we first converted the gene ID from the Official Symbol of the differential gene obtained by org.Hs.eg. Db package [36] and clusterProfiler package [37] were adopted for GO and KEGG pathway analysis, respectively. Besides, the GOplot package [38] was used for cluster analysis. Adj. *p*. Val <0.05 denoted statistical significance.

### MCODE analysis

Differentially expressed genes (DEGs) were analyzed using Metascape online database (http://metascape.org/gp/index.html#/main/step1) [39]. KEGG pathway enrichment analysis was performed for each MCODE. *P* < 0.01 denoted statistical significance.

### PPI network construction and hub gene screening

STRING [40] database (https://string-db.org/) was used to construct a PPI network. An interaction with a composite score greater than 0.9 was considered statistically significant. Cytoscape [41] is an open-source bioinformatics software program for exploring molecular interaction networks. CytoHubba [42], a Cytoscape Plugin, was used to evaluate PPI network hub genes. The top 20 hub genes were screened out through CytoHubba Plugin by degree. Red colors denoted that the gene had a higher degree.

### Bioinformatics analysis of hub gene

The top 20 hub Genes were analyzed via Metascape online database (http://metascape.org/gp/index.html#/main/step1). Gene list enrichments were identified in the following ontology categories: TRRUST, DisGeNET, and PaGenBase. All genes in the genome were used as the enrichment background. Terms with a *p*-value < 0.01, a minimum count of 3, and an enrichment factor > 1.5 were screened out. Interaction of small-molecule drugs with hub genes was predicted using CMAP online database (https://portals.broadinstitute.org/cmap).

## Supplementary Materials

Supplementary Table 1
