# Supplementary material for: Identification of key pathways and genes in carotid atherosclerosis through bioinformatics analysis of RNA-seq data
Source: Aging (Albany NY). 2021 May 11;13(9):12733–47. doi: 10.18632/aging.202943 (PMC8148499; doi:10.18632/aging.202943)
Supplement: Supplementary Table 1 [file aging-13-202943-s001.doc]

| **Supplementary Table 1. Identification of differentially expressed genes.** | | | | |
| --- | --- | --- | --- | --- |
| **gene_id** | **gene_name** | **log2FC** | **pvalue** | **adj.p.Val** |
| ENSG00000189058 | APOD | -6.223684829 | 1.15E-73 | 2.58E-69 |
| ENSG00000169418 | NPR1 | -4.454295897 | 1.33E-71 | 1.49E-67 |
| ENSG00000173641 | HSPB7 | -4.303132926 | 4.95E-61 | 3.71E-57 |
| ENSG00000262655 | SPON1 | -5.02174084 | 8.88E-50 | 4.99E-46 |
| ENSG00000188257 | PLA2G2A | -12.21395477 | 2.87E-47 | 1.29E-43 |
| ENSG00000173175 | ADCY5 | -3.700608709 | 4.52E-42 | 1.70E-38 |
| ENSG00000168079 | SCARA5 | -6.170052783 | 2.61E-41 | 8.40E-38 |
| ENSG00000197380 | DACT3 | -3.885226278 | 9.73E-38 | 2.73E-34 |
| ENSG00000018236 | CNTN1 | -6.608542113 | 2.43E-37 | 6.07E-34 |
| ENSG00000111863 | ADTRP | 5.369454297 | 4.51E-37 | 1.02E-33 |
| ENSG00000103710 | RASL12 | -3.942339742 | 1.09E-35 | 2.23E-32 |
| ENSG00000018625 | ATP1A2 | -5.246926737 | 7.93E-35 | 1.49E-31 |
| ENSG00000143248 | RGS5 | -3.408524754 | 1.16E-34 | 2.01E-31 |
| ENSG00000144857 | BOC | -4.009780331 | 6.12E-34 | 9.82E-31 |
| ENSG00000173705 | SUSD5 | -3.189978483 | 9.83E-34 | 1.47E-30 |
| ENSG00000261150 | EPPK1 | -4.90274095 | 1.12E-33 | 1.57E-30 |
| ENSG00000168743 | NPNT | -3.249646247 | 2.79E-33 | 3.69E-30 |
| ENSG00000149596 | JPH2 | -3.497226217 | 4.10E-33 | 5.13E-30 |
| ENSG00000122367 | LDB3 | -3.762971439 | 4.57E-33 | 5.41E-30 |
| ENSG00000149451 | ADAM33 | -3.701378947 | 2.78E-32 | 3.13E-29 |
| ENSG00000128591 | FLNC | -4.956058889 | 9.06E-32 | 9.70E-29 |
| ENSG00000175084 | DES | -6.140234842 | 1.22E-31 | 1.25E-28 |
| ENSG00000184160 | ADRA2C | -6.184388137 | 2.97E-31 | 2.90E-28 |
| ENSG00000072195 | SPEG | -3.239927002 | 4.14E-31 | 3.88E-28 |
| ENSG00000197506 | SLC28A3 | 7.144802462 | 1.23E-29 | 1.11E-26 |
| ENSG00000159251 | ACTC1 | -5.065301653 | 1.29E-29 | 1.11E-26 |
| ENSG00000172935 | MRGPRF | -3.240832766 | 9.29E-29 | 7.46E-26 |
| ENSG00000106018 | VIPR2 | -4.203225097 | 1.46E-28 | 1.13E-25 |
| ENSG00000112394 | SLC16A10 | 3.755627982 | 6.45E-28 | 4.83E-25 |
| ENSG00000183963 | SMTN | -3.334568098 | 9.04E-28 | 6.55E-25 |
| ENSG00000101230 | ISM1 | -4.265086094 | 8.10E-27 | 5.52E-24 |
| ENSG00000132938 | MTUS2 | -5.002501124 | 1.75E-26 | 1.16E-23 |
| ENSG00000124107 | SLPI | -3.959637842 | 3.75E-26 | 2.41E-23 |
| ENSG00000182195 | LDOC1 | -3.835379186 | 4.47E-26 | 2.79E-23 |
| ENSG00000168490 | PHYHIP | -4.279402101 | 5.71E-26 | 3.47E-23 |
| ENSG00000165912 | PACSIN3 | -3.382792201 | 8.96E-26 | 5.30E-23 |
| ENSG00000135424 | ITGA7 | -3.453162629 | 2.46E-25 | 1.42E-22 |
| ENSG00000124731 | TREM1 | 4.94291838 | 6.95E-25 | 3.89E-22 |
| ENSG00000205363 | INSYN1 | -4.209088986 | 7.84E-25 | 4.20E-22 |
| ENSG00000121933 | TMIGD3 | 4.250011789 | 9.09E-25 | 4.75E-22 |
| ENSG00000068615 | REEP1 | -4.972404757 | 1.35E-24 | 6.59E-22 |
| ENSG00000180287 | PLD5 | -6.900468692 | 1.76E-24 | 8.43E-22 |
| ENSG00000174808 | BTC | -4.534749652 | 2.05E-24 | 9.59E-22 |
| ENSG00000072694 | FCGR2B | 3.882808202 | 4.73E-24 | 2.17E-21 |
| ENSG00000130176 | CNN1 | -3.982279465 | 7.12E-24 | 3.20E-21 |
| ENSG00000125355 | TMEM255A | 4.637414866 | 7.76E-24 | 3.42E-21 |
| ENSG00000143867 | OSR1 | -3.123424536 | 9.78E-24 | 4.23E-21 |
| ENSG00000076706 | MCAM | -3.58999875 | 1.11E-23 | 4.70E-21 |
| ENSG00000187134 | AKR1C1 | -3.365054248 | 1.45E-23 | 6.05E-21 |
| ENSG00000244682 | FCGR2C | 4.132029155 | 1.64E-23 | 6.71E-21 |
| ENSG00000066468 | FGFR2 | -5.736092202 | 1.75E-23 | 7.01E-21 |
| ENSG00000100628 | ASB2 | -3.34471255 | 1.89E-23 | 7.47E-21 |
| ENSG00000152078 | TMEM56 | -3.297366834 | 3.12E-23 | 1.21E-20 |
| ENSG00000065320 | NTN1 | -4.412487356 | 3.57E-23 | 1.36E-20 |
| ENSG00000131831 | RAI2 | -3.121264018 | 3.96E-23 | 1.48E-20 |
| ENSG00000153707 | PTPRD | -3.972998542 | 5.93E-23 | 2.19E-20 |
| ENSG00000117013 | KCNQ4 | -3.343207314 | 1.35E-22 | 4.88E-20 |
| ENSG00000164764 | SBSPON | -4.641426682 | 2.11E-22 | 7.43E-20 |
| ENSG00000132329 | RAMP1 | -3.487115351 | 2.98E-22 | 1.03E-19 |
| ENSG00000146555 | SDK1 | -3.787706641 | 3.14E-22 | 1.06E-19 |
| ENSG00000145824 | CXCL14 | -10.1048008 | 3.16E-22 | 1.06E-19 |
| ENSG00000280237 | MIR4697HG | -3.63624243 | 1.71E-21 | 5.49E-19 |
| ENSG00000146197 | SCUBE3 | -3.980746646 | 1.75E-21 | 5.55E-19 |
| ENSG00000164530 | PI16 | -5.275488363 | 1.79E-21 | 5.57E-19 |
| ENSG00000034971 | MYOC | -7.057547407 | 1.81E-21 | 5.57E-19 |
| ENSG00000121361 | KCNJ8 | -3.84833211 | 1.84E-21 | 5.59E-19 |
| ENSG00000158258 | CLSTN2 | -6.507621502 | 2.20E-21 | 6.60E-19 |
| ENSG00000257017 | HP | -7.326744147 | 2.80E-21 | 8.29E-19 |
| ENSG00000182175 | RGMA | -3.342281662 | 3.68E-21 | 1.08E-18 |
| ENSG00000121440 | PDZRN3 | -3.585954098 | 4.45E-21 | 1.28E-18 |
| ENSG00000165996 | HACD1 | -3.231093923 | 5.43E-21 | 1.55E-18 |
| ENSG00000225217 | HSPA7 | 4.120530324 | 6.45E-21 | 1.81E-18 |
| ENSG00000142621 | FHAD1 | 4.613552324 | 1.54E-20 | 4.28E-18 |
| ENSG00000182253 | SYNM | -3.001247362 | 1.69E-20 | 4.64E-18 |
| ENSG00000169181 | GSG1L | -5.768455063 | 2.08E-20 | 5.61E-18 |
| ENSG00000115361 | ACADL | -3.96539716 | 2.09E-20 | 5.61E-18 |
| ENSG00000181885 | CLDN7 | 3.529942037 | 2.74E-20 | 7.25E-18 |
| ENSG00000184905 | TCEAL2 | -4.893730444 | 4.10E-20 | 1.07E-17 |
| ENSG00000108830 | RND2 | -4.743810495 | 4.71E-20 | 1.22E-17 |
| ENSG00000018280 | SLC11A1 | 4.368746292 | 5.07E-20 | 1.29E-17 |
| ENSG00000169184 | MN1 | -3.584851217 | 6.24E-20 | 1.58E-17 |
| ENSG00000221890 | NPTXR | -3.428564538 | 6.95E-20 | 1.74E-17 |
| ENSG00000116141 | MARK1 | -3.77804373 | 7.29E-20 | 1.78E-17 |
| ENSG00000099994 | SUSD2 | -3.827010986 | 7.39E-20 | 1.78E-17 |
| ENSG00000006747 | SCIN | 4.501205095 | 7.44E-20 | 1.78E-17 |
| ENSG00000126878 | AIF1L | -3.322431007 | 8.67E-20 | 2.05E-17 |
| ENSG00000101938 | CHRDL1 | -4.707685294 | 1.17E-19 | 2.71E-17 |
| ENSG00000147180 | ZNF711 | -3.252336685 | 1.28E-19 | 2.94E-17 |
| ENSG00000198842 | DUSP27 | -4.089896076 | 1.39E-19 | 3.12E-17 |
| ENSG00000118785 | SPP1 | 6.479653343 | 1.45E-19 | 3.22E-17 |
| ENSG00000135116 | HRK | 5.51373247 | 1.94E-19 | 4.23E-17 |
| ENSG00000139364 | TMEM132B | -5.072149754 | 2.19E-19 | 4.73E-17 |
| ENSG00000124191 | TOX2 | -4.02572949 | 2.31E-19 | 4.95E-17 |
| ENSG00000143217 | NECTIN4 | 6.525819533 | 2.48E-19 | 5.25E-17 |
| ENSG00000142611 | PRDM16 | -3.040565268 | 3.14E-19 | 6.59E-17 |
| ENSG00000170801 | HTRA3 | -4.191584463 | 4.44E-19 | 9.24E-17 |
| ENSG00000136689 | IL1RN | 6.188469432 | 4.56E-19 | 9.41E-17 |
| ENSG00000133878 | DUSP26 | -3.770052543 | 5.65E-19 | 1.15E-16 |
| ENSG00000108688 | CCL7 | 8.011386733 | 6.44E-19 | 1.29E-16 |
| ENSG00000153446 | C16orf89 | -5.65477932 | 8.43E-19 | 1.68E-16 |
| ENSG00000187068 | C3orf70 | -3.640796769 | 1.03E-18 | 2.01E-16 |
| ENSG00000182575 | NXPH3 | -3.437642222 | 1.56E-18 | 2.98E-16 |
| ENSG00000115041 | KCNIP3 | -4.688076122 | 1.88E-18 | 3.56E-16 |
| ENSG00000153531 | ADPRHL1 | -4.545139457 | 2.11E-18 | 3.96E-16 |
| ENSG00000166831 | RBPMS2 | -3.862089394 | 2.32E-18 | 4.32E-16 |
| ENSG00000119686 | FLVCR2 | 3.491050777 | 2.93E-18 | 5.39E-16 |
| ENSG00000163431 | LMOD1 | -3.094496108 | 3.01E-18 | 5.51E-16 |
| ENSG00000224397 | SMIM25 | 4.955651149 | 3.07E-18 | 5.58E-16 |
| ENSG00000171729 | TMEM51 | 3.631524614 | 3.33E-18 | 6.00E-16 |
| ENSG00000150394 | CDH8 | -5.720511365 | 3.41E-18 | 6.08E-16 |
| ENSG00000101307 | SIRPB1 | 5.979629275 | 3.74E-18 | 6.62E-16 |
| ENSG00000165124 | SVEP1 | -4.203801697 | 5.58E-18 | 9.72E-16 |
| ENSG00000118113 | MMP8 | 10.7185406 | 5.64E-18 | 9.75E-16 |
| ENSG00000138316 | ADAMTS14 | 3.067171099 | 6.04E-18 | 1.04E-15 |
| ENSG00000138771 | SHROOM3 | -3.042896775 | 1.77E-17 | 3.01E-15 |
| ENSG00000182612 | TSPAN10 | 5.441973155 | 2.77E-17 | 4.59E-15 |
| ENSG00000124780 | KCNK17 | -4.99409932 | 2.92E-17 | 4.79E-15 |
| ENSG00000198814 | GK | 3.822992288 | 3.28E-17 | 5.34E-15 |
| ENSG00000103196 | CRISPLD2 | -4.263520516 | 3.31E-17 | 5.35E-15 |
| ENSG00000185274 | GALNT17 | -4.256416814 | 3.51E-17 | 5.60E-15 |
| ENSG00000187479 | C11orf96 | -3.401080607 | 3.76E-17 | 5.95E-15 |
| ENSG00000125675 | GRIA3 | -4.117659227 | 4.11E-17 | 6.46E-15 |
| ENSG00000172061 | LRRC15 | 5.392229049 | 4.22E-17 | 6.59E-15 |
| ENSG00000260604 | AL590004.3 | -3.861025132 | 5.58E-17 | 8.65E-15 |
| ENSG00000077522 | ACTN2 | -4.425381983 | 5.64E-17 | 8.69E-15 |
| ENSG00000151651 | ADAM8 | 5.70009105 | 7.46E-17 | 1.13E-14 |
| ENSG00000144619 | CNTN4 | -4.507874733 | 8.28E-17 | 1.25E-14 |
| ENSG00000267452 | LINC02073 | 7.840703272 | 8.96E-17 | 1.34E-14 |
| ENSG00000150337 | FCGR1A | 3.440604973 | 9.30E-17 | 1.38E-14 |
| ENSG00000104332 | SFRP1 | -6.497077628 | 1.02E-16 | 1.51E-14 |
| ENSG00000152583 | SPARCL1 | -3.694799937 | 1.03E-16 | 1.51E-14 |
| ENSG00000130037 | KCNA5 | -4.328338922 | 1.08E-16 | 1.58E-14 |
| ENSG00000166928 | MS4A14 | 3.831405321 | 1.15E-16 | 1.66E-14 |
| ENSG00000151789 | ZNF385D | -3.076007779 | 1.44E-16 | 2.07E-14 |
| ENSG00000101160 | CTSZ | 3.622423583 | 1.64E-16 | 2.32E-14 |
| ENSG00000198523 | PLN | -3.057363959 | 1.69E-16 | 2.35E-14 |
| ENSG00000104951 | IL4I1 | 4.743372545 | 1.71E-16 | 2.37E-14 |
| ENSG00000170909 | OSCAR | 4.564187316 | 1.72E-16 | 2.37E-14 |
| ENSG00000130558 | OLFM1 | -3.490086831 | 1.90E-16 | 2.60E-14 |
| ENSG00000184343 | SRPK3 | -3.319945139 | 1.95E-16 | 2.66E-14 |
| ENSG00000133067 | LGR6 | -4.144264565 | 1.97E-16 | 2.67E-14 |
| ENSG00000182916 | TCEAL7 | -3.780618265 | 2.27E-16 | 3.05E-14 |
| ENSG00000109576 | AADAT | -3.199770468 | 2.85E-16 | 3.79E-14 |
| ENSG00000258227 | CLEC5A | 3.669396028 | 3.05E-16 | 4.04E-14 |
| ENSG00000171873 | ADRA1D | -7.945438964 | 3.16E-16 | 4.16E-14 |
| ENSG00000262580 | AC087741.1 | 3.453118163 | 3.45E-16 | 4.51E-14 |
| ENSG00000118898 | PPL | -4.576146234 | 4.79E-16 | 6.22E-14 |
| ENSG00000100292 | HMOX1 | 3.036430564 | 6.04E-16 | 7.80E-14 |
| ENSG00000101333 | PLCB4 | -3.962075934 | 7.39E-16 | 9.45E-14 |
| ENSG00000174807 | CD248 | -3.690256657 | 7.98E-16 | 1.01E-13 |
| ENSG00000180447 | GAS1 | -3.601023393 | 8.13E-16 | 1.03E-13 |
| ENSG00000159164 | SV2A | -3.10305391 | 8.34E-16 | 1.05E-13 |
| ENSG00000204103 | MAFB | 3.002490351 | 8.55E-16 | 1.07E-13 |
| ENSG00000164106 | SCRG1 | -4.620655675 | 1.57E-15 | 1.92E-13 |
| ENSG00000154734 | ADAMTS1 | -3.464744549 | 1.68E-15 | 2.02E-13 |
| ENSG00000277531 | PNMA8C | -3.571136694 | 1.69E-15 | 2.02E-13 |
| ENSG00000198246 | SLC29A3 | 3.960332326 | 1.69E-15 | 2.02E-13 |
| ENSG00000169495 | HTRA4 | 6.089020841 | 1.76E-15 | 2.08E-13 |
| ENSG00000175928 | LRRN1 | -5.744658807 | 2.09E-15 | 2.45E-13 |
| ENSG00000101098 | RIMS4 | -4.359333632 | 2.64E-15 | 3.04E-13 |
| ENSG00000204065 | TCEAL5 | -5.950245547 | 2.65E-15 | 3.04E-13 |
| ENSG00000166278 | C2 | 3.984185918 | 2.95E-15 | 3.37E-13 |
| ENSG00000163884 | KLF15 | -3.085146891 | 3.01E-15 | 3.41E-13 |
| ENSG00000196616 | ADH1B | -3.329106578 | 3.89E-15 | 4.33E-13 |
| ENSG00000170500 | LONRF2 | -3.7811895 | 4.00E-15 | 4.44E-13 |
| ENSG00000164128 | NPY1R | -3.446142597 | 4.11E-15 | 4.53E-13 |
| ENSG00000141526 | SLC16A3 | 3.064324307 | 4.46E-15 | 4.89E-13 |
| ENSG00000136235 | GPNMB | 3.829691604 | 4.66E-15 | 5.09E-13 |
| ENSG00000143028 | SYPL2 | -3.342277045 | 4.88E-15 | 5.30E-13 |
| ENSG00000198626 | RYR2 | -4.531131232 | 4.97E-15 | 5.38E-13 |
| ENSG00000140092 | FBLN5 | -3.081315875 | 5.59E-15 | 5.95E-13 |
| ENSG00000135324 | MRAP2 | -4.184122234 | 6.02E-15 | 6.35E-13 |
| ENSG00000151892 | GFRA1 | -11.92364524 | 6.88E-15 | 7.20E-13 |
| ENSG00000144891 | AGTR1 | -7.233142484 | 7.22E-15 | 7.52E-13 |
| ENSG00000108551 | RASD1 | -4.227283193 | 7.36E-15 | 7.63E-13 |
| ENSG00000100302 | RASD2 | -5.530925087 | 8.10E-15 | 8.32E-13 |
| ENSG00000164946 | FREM1 | -4.529102336 | 1.02E-14 | 1.03E-12 |
| ENSG00000113805 | CNTN3 | -3.143589741 | 1.09E-14 | 1.09E-12 |
| ENSG00000155465 | SLC7A7 | 3.252787751 | 1.45E-14 | 1.44E-12 |
| ENSG00000068976 | PYGM | -3.102738424 | 1.70E-14 | 1.65E-12 |
| ENSG00000117984 | CTSD | 3.951596885 | 1.85E-14 | 1.79E-12 |
| ENSG00000158104 | HPD | -3.120274372 | 1.87E-14 | 1.79E-12 |
| ENSG00000170439 | METTL7B | 3.548123124 | 2.27E-14 | 2.17E-12 |
| ENSG00000172987 | HPSE2 | -6.478992009 | 3.47E-14 | 3.29E-12 |
| ENSG00000137673 | MMP7 | 13.62598352 | 4.05E-14 | 3.81E-12 |
| ENSG00000186047 | DLEU7 | 4.035697532 | 4.09E-14 | 3.83E-12 |
| ENSG00000144218 | AFF3 | -3.694960547 | 4.27E-14 | 3.97E-12 |
| ENSG00000123560 | PLP1 | -4.717664392 | 4.27E-14 | 3.97E-12 |
| ENSG00000244482 | LILRA6 | 3.874463734 | 4.67E-14 | 4.32E-12 |
| ENSG00000169896 | ITGAM | 3.792978338 | 5.13E-14 | 4.70E-12 |
| ENSG00000107593 | PKD2L1 | 5.608257715 | 5.14E-14 | 4.70E-12 |
| ENSG00000244953 | AC087521.1 | -4.049486229 | 5.29E-14 | 4.79E-12 |
| ENSG00000153993 | SEMA3D | -4.49117357 | 5.66E-14 | 5.05E-12 |
| ENSG00000154263 | ABCA10 | -5.447461997 | 6.86E-14 | 6.00E-12 |
| ENSG00000153976 | HS3ST3A1 | 4.53056041 | 7.04E-14 | 6.14E-12 |
| ENSG00000149418 | ST14 | 5.49464763 | 7.52E-14 | 6.53E-12 |
| ENSG00000026297 | RNASET2 | 3.809247099 | 7.64E-14 | 6.61E-12 |
| ENSG00000141639 | MAPK4 | -7.895837872 | 9.79E-14 | 8.34E-12 |
| ENSG00000162520 | SYNC | -3.391982609 | 1.05E-13 | 8.89E-12 |
| ENSG00000140379 | BCL2A1 | 5.017277339 | 1.16E-13 | 9.73E-12 |
| ENSG00000261625 | AP003071.4 | -4.374773477 | 1.20E-13 | 9.99E-12 |
| ENSG00000224137 | LINC01857 | 5.958970124 | 1.20E-13 | 9.99E-12 |
| ENSG00000168077 | SCARA3 | -3.08816101 | 1.37E-13 | 1.13E-11 |
| ENSG00000106772 | PRUNE2 | -3.057089351 | 1.51E-13 | 1.25E-11 |
| ENSG00000111199 | TRPV4 | 3.130000073 | 1.78E-13 | 1.45E-11 |
| ENSG00000154856 | APCDD1 | -3.058590212 | 1.90E-13 | 1.54E-11 |
| ENSG00000167676 | PLIN4 | -6.22624428 | 2.20E-13 | 1.76E-11 |
| ENSG00000249001 | AC093895.1 | 5.194301811 | 2.61E-13 | 2.06E-11 |
| ENSG00000073060 | SCARB1 | 3.15624618 | 2.93E-13 | 2.30E-11 |
| ENSG00000144681 | STAC | -3.909349836 | 3.13E-13 | 2.44E-11 |
| ENSG00000069188 | SDK2 | -3.882250942 | 3.37E-13 | 2.61E-11 |
| ENSG00000156103 | MMP16 | -3.923138618 | 3.56E-13 | 2.75E-11 |
| ENSG00000141052 | MYOCD | -4.335328912 | 3.66E-13 | 2.81E-11 |
| ENSG00000091106 | NLRC4 | 3.241344511 | 3.67E-13 | 2.81E-11 |
| ENSG00000105357 | MYH14 | -3.499414416 | 3.87E-13 | 2.94E-11 |
| ENSG00000170214 | ADRA1B | -3.906830858 | 4.03E-13 | 3.05E-11 |
| ENSG00000171724 | VAT1L | -4.527408908 | 4.55E-13 | 3.38E-11 |
| ENSG00000077585 | GPR137B | 3.183605422 | 4.85E-13 | 3.60E-11 |
| ENSG00000135312 | HTR1B | -3.06852074 | 4.87E-13 | 3.60E-11 |
| ENSG00000205221 | VIT | -11.27539068 | 6.57E-13 | 4.80E-11 |
| ENSG00000143318 | CASQ1 | -5.98490161 | 7.02E-13 | 5.09E-11 |
| ENSG00000109846 | CRYAB | -3.400802867 | 8.18E-13 | 5.88E-11 |
| ENSG00000155265 | GOLGA7B | 4.486131968 | 8.69E-13 | 6.22E-11 |
| ENSG00000104783 | KCNN4 | 3.490787648 | 8.86E-13 | 6.32E-11 |
| ENSG00000163017 | ACTG2 | -3.598407942 | 8.90E-13 | 6.33E-11 |
| ENSG00000262406 | MMP12 | 11.54415798 | 9.72E-13 | 6.90E-11 |
| ENSG00000140807 | NKD1 | -3.387547122 | 1.00E-12 | 7.08E-11 |
| ENSG00000110446 | SLC15A3 | 3.18249521 | 1.05E-12 | 7.39E-11 |
| ENSG00000177133 | LINC00982 | -3.583474824 | 1.12E-12 | 7.82E-11 |
| ENSG00000180525 | PRR26 | -3.275214099 | 1.13E-12 | 7.85E-11 |
| ENSG00000066382 | MPPED2 | -3.320059514 | 1.22E-12 | 8.46E-11 |
| ENSG00000152822 | GRM1 | 4.91717735 | 1.37E-12 | 9.45E-11 |
| ENSG00000142973 | CYP4B1 | -11.24280191 | 1.41E-12 | 9.63E-11 |
| ENSG00000164920 | OSR2 | -4.426066632 | 1.45E-12 | 9.87E-11 |
| ENSG00000078053 | AMPH | -4.314409422 | 1.60E-12 | 1.08E-10 |
| ENSG00000103647 | CORO2B | -3.551553578 | 1.69E-12 | 1.13E-10 |
| ENSG00000186198 | SLC51B | -4.086513219 | 1.74E-12 | 1.16E-10 |
| ENSG00000011422 | PLAUR | 3.26324662 | 2.00E-12 | 1.33E-10 |
| ENSG00000146094 | DOK3 | 3.516615539 | 2.12E-12 | 1.40E-10 |
| ENSG00000219438 | FAM19A5 | -4.160143705 | 2.62E-12 | 1.70E-10 |
| ENSG00000075275 | CELSR1 | 4.253547285 | 3.05E-12 | 1.93E-10 |
| ENSG00000276085 | CCL3L1 | 3.433709969 | 3.14E-12 | 1.98E-10 |
| ENSG00000172243 | CLEC7A | 3.058830916 | 3.25E-12 | 2.04E-10 |
| ENSG00000062282 | DGAT2 | -5.304406269 | 3.37E-12 | 2.11E-10 |
| ENSG00000042493 | CAPG | 4.398608523 | 3.61E-12 | 2.23E-10 |
| ENSG00000095383 | TBC1D2 | 3.153142035 | 3.61E-12 | 2.23E-10 |
| ENSG00000259953 | AL138756.1 | 3.109010091 | 3.71E-12 | 2.29E-10 |
| ENSG00000166145 | SPINT1 | 3.068288096 | 3.87E-12 | 2.36E-10 |
| ENSG00000112799 | LY86 | 3.994036286 | 4.15E-12 | 2.53E-10 |
| ENSG00000088320 | REM1 | -3.272769605 | 4.18E-12 | 2.54E-10 |
| ENSG00000239998 | LILRA2 | 3.373863375 | 4.43E-12 | 2.67E-10 |
| ENSG00000095739 | BAMBI | -3.28001922 | 4.71E-12 | 2.82E-10 |
| ENSG00000171611 | PTCRA | 4.993036217 | 4.76E-12 | 2.84E-10 |
| ENSG00000133392 | MYH11 | -3.021418966 | 5.11E-12 | 3.03E-10 |
| ENSG00000172594 | SMPDL3A | 3.693850851 | 5.46E-12 | 3.23E-10 |
| ENSG00000188060 | RAB42 | 4.303319072 | 5.55E-12 | 3.27E-10 |
| ENSG00000121594 | CD80 | 3.623440725 | 5.91E-12 | 3.46E-10 |
| ENSG00000138356 | AOX1 | -4.046260564 | 6.29E-12 | 3.65E-10 |
| ENSG00000111181 | SLC6A12 | 3.598994169 | 6.44E-12 | 3.73E-10 |
| ENSG00000156475 | PPP2R2B | -3.222277383 | 6.99E-12 | 4.03E-10 |
| ENSG00000164733 | CTSB | 4.360834395 | 6.99E-12 | 4.03E-10 |
| ENSG00000148053 | NTRK2 | -3.225799353 | 7.34E-12 | 4.19E-10 |
| ENSG00000143786 | CNIH3 | 3.844156339 | 7.82E-12 | 4.44E-10 |
| ENSG00000108700 | CCL8 | 3.613118363 | 1.11E-11 | 6.14E-10 |
| ENSG00000104972 | LILRB1 | 3.553057615 | 1.24E-11 | 6.82E-10 |
| ENSG00000169760 | NLGN1 | -3.166596551 | 1.24E-11 | 6.82E-10 |
| ENSG00000184226 | PCDH9 | -5.04536884 | 1.35E-11 | 7.36E-10 |
| ENSG00000180061 | TMEM150B | 3.961020127 | 1.40E-11 | 7.64E-10 |
| ENSG00000242265 | PEG10 | -3.822748246 | 1.71E-11 | 9.26E-10 |
| ENSG00000008118 | CAMK1G | 4.310912922 | 1.75E-11 | 9.42E-10 |
| ENSG00000170396 | ZNF804A | 4.662300829 | 1.77E-11 | 9.50E-10 |
| ENSG00000172399 | MYOZ2 | -3.002598134 | 1.80E-11 | 9.60E-10 |
| ENSG00000181856 | SLC2A4 | -3.911469373 | 1.80E-11 | 9.60E-10 |
| ENSG00000197993 | KEL | 5.392915751 | 2.03E-11 | 1.08E-09 |
| ENSG00000144229 | THSD7B | -4.340132794 | 2.08E-11 | 1.10E-09 |
| ENSG00000224307 | AL161785.1 | 3.232372262 | 2.08E-11 | 1.10E-09 |
| ENSG00000198838 | RYR3 | -3.509199641 | 2.11E-11 | 1.11E-09 |
| ENSG00000179163 | FUCA1 | 3.622273549 | 2.22E-11 | 1.15E-09 |
| ENSG00000132205 | EMILIN2 | 3.205276044 | 2.27E-11 | 1.17E-09 |
| ENSG00000254535 | PABPC4L | -3.197055396 | 2.28E-11 | 1.17E-09 |
| ENSG00000022556 | NLRP2 | 4.606532841 | 2.35E-11 | 1.21E-09 |
| ENSG00000066336 | SPI1 | 3.17388488 | 2.36E-11 | 1.21E-09 |
| ENSG00000144908 | ALDH1L1 | -3.458236255 | 2.37E-11 | 1.22E-09 |
| ENSG00000154645 | CHODL | -7.717793428 | 2.49E-11 | 1.27E-09 |
| ENSG00000126950 | TMEM35A | -3.772121231 | 2.55E-11 | 1.30E-09 |
| ENSG00000277632 | CCL3 | 4.48454788 | 2.74E-11 | 1.38E-09 |
| ENSG00000116661 | FBXO2 | -4.417432948 | 2.82E-11 | 1.42E-09 |
| ENSG00000156711 | MAPK13 | 3.724981413 | 2.91E-11 | 1.46E-09 |
| ENSG00000121904 | CSMD2 | -3.555048527 | 2.92E-11 | 1.46E-09 |
| ENSG00000167851 | CD300A | 4.086064422 | 2.93E-11 | 1.46E-09 |
| ENSG00000101605 | MYOM1 | -3.256293105 | 3.22E-11 | 1.60E-09 |
| ENSG00000221818 | EBF2 | -4.633854606 | 3.28E-11 | 1.63E-09 |
| ENSG00000135929 | CYP27A1 | 3.284017804 | 3.60E-11 | 1.77E-09 |
| ENSG00000146267 | FAXC | -4.849674754 | 3.61E-11 | 1.77E-09 |
| ENSG00000106236 | NPTX2 | -4.427439105 | 3.84E-11 | 1.87E-09 |
| ENSG00000110031 | LPXN | 3.295566581 | 4.36E-11 | 2.10E-09 |
| ENSG00000115009 | CCL20 | 5.795175522 | 4.57E-11 | 2.19E-09 |
| ENSG00000139629 | GALNT6 | 3.931948778 | 5.26E-11 | 2.51E-09 |
| ENSG00000196743 | GM2A | 3.620230197 | 5.52E-11 | 2.62E-09 |
| ENSG00000000938 | FGR | 3.795459704 | 5.65E-11 | 2.67E-09 |
| ENSG00000203805 | PLPP4 | 4.513401174 | 5.69E-11 | 2.68E-09 |
| ENSG00000158246 | TENT5B | -3.331821549 | 5.70E-11 | 2.68E-09 |
| ENSG00000131095 | GFAP | -4.664507254 | 5.95E-11 | 2.79E-09 |
| ENSG00000157150 | TIMP4 | -3.248258696 | 6.04E-11 | 2.82E-09 |
| ENSG00000162545 | CAMK2N1 | -3.076785185 | 6.12E-11 | 2.84E-09 |
| ENSG00000229512 | AC068580.1 | 4.236786866 | 6.42E-11 | 2.95E-09 |
| ENSG00000118729 | CASQ2 | -4.456451617 | 6.85E-11 | 3.13E-09 |
| ENSG00000030582 | GRN | 3.092878775 | 7.03E-11 | 3.20E-09 |
| ENSG00000118242 | MREG | 4.851793695 | 7.14E-11 | 3.25E-09 |
| ENSG00000168779 | SHOX2 | 3.183757719 | 7.40E-11 | 3.36E-09 |
| ENSG00000162738 | VANGL2 | -3.790759797 | 7.69E-11 | 3.47E-09 |
| ENSG00000187715 | KBTBD12 | -3.39291617 | 7.97E-11 | 3.58E-09 |
| ENSG00000135838 | NPL | 4.196727558 | 9.36E-11 | 4.16E-09 |
| ENSG00000064300 | NGFR | -3.965079643 | 1.01E-10 | 4.44E-09 |
| ENSG00000116194 | ANGPTL1 | -3.944940687 | 1.02E-10 | 4.48E-09 |
| ENSG00000100600 | LGMN | 3.481648918 | 1.02E-10 | 4.50E-09 |
| ENSG00000245848 | CEBPA | 3.437618778 | 1.04E-10 | 4.55E-09 |
| ENSG00000163823 | CCR1 | 3.320647771 | 1.05E-10 | 4.57E-09 |
| ENSG00000108231 | LGI1 | -4.173148009 | 1.08E-10 | 4.69E-09 |
| ENSG00000197249 | SERPINA1 | 4.135175535 | 1.10E-10 | 4.77E-09 |
| ENSG00000211640 | IGLV6-57 | 4.197775682 | 1.19E-10 | 5.16E-09 |
| ENSG00000164687 | FABP5 | 3.559812349 | 1.23E-10 | 5.29E-09 |
| ENSG00000204577 | LILRB3 | 3.511391843 | 1.28E-10 | 5.47E-09 |
| ENSG00000175489 | LRRC25 | 3.223668384 | 1.31E-10 | 5.60E-09 |
| ENSG00000107551 | RASSF4 | 3.119685125 | 1.37E-10 | 5.84E-09 |
| ENSG00000162511 | LAPTM5 | 3.910102071 | 1.47E-10 | 6.26E-09 |
| ENSG00000171227 | TMEM37 | 3.161849773 | 1.47E-10 | 6.26E-09 |
| ENSG00000154639 | CXADR | 4.80152061 | 1.54E-10 | 6.53E-09 |
| ENSG00000136286 | MYO1G | 3.632046734 | 1.67E-10 | 6.99E-09 |
| ENSG00000100558 | PLEK2 | 3.879411911 | 1.68E-10 | 6.99E-09 |
| ENSG00000112299 | VNN1 | 3.646402219 | 1.75E-10 | 7.28E-09 |
| ENSG00000101197 | BIRC7 | 5.349687617 | 1.83E-10 | 7.59E-09 |
| ENSG00000112276 | BVES | -3.461364087 | 1.84E-10 | 7.59E-09 |
| ENSG00000117009 | KMO | 4.354452732 | 1.90E-10 | 7.82E-09 |
| ENSG00000164509 | IL31RA | -4.801576148 | 1.95E-10 | 8.00E-09 |
| ENSG00000105383 | CD33 | 3.106555531 | 2.07E-10 | 8.44E-09 |
| ENSG00000255422 | AP002954.1 | 4.101085141 | 2.29E-10 | 9.24E-09 |
| ENSG00000158528 | PPP1R9A | -4.116003741 | 2.33E-10 | 9.42E-09 |
| ENSG00000177807 | KCNJ10 | 4.070627821 | 2.43E-10 | 9.80E-09 |
| ENSG00000085514 | PILRA | 3.669634839 | 2.63E-10 | 1.05E-08 |
| ENSG00000251442 | LINC01094 | 3.889900513 | 2.97E-10 | 1.18E-08 |
| ENSG00000136295 | TTYH3 | 3.108061065 | 3.13E-10 | 1.24E-08 |
| ENSG00000261655 | AC100803.2 | -4.218959093 | 3.16E-10 | 1.25E-08 |
| ENSG00000128262 | POM121L9P | -3.647349725 | 3.33E-10 | 1.31E-08 |
| ENSG00000128815 | WDFY4 | 3.731372441 | 3.43E-10 | 1.34E-08 |
| ENSG00000137261 | KIAA0319 | 5.26716604 | 3.46E-10 | 1.35E-08 |
| ENSG00000019169 | MARCO | 3.990941518 | 3.47E-10 | 1.36E-08 |
| ENSG00000253636 | AC022893.1 | -3.363100675 | 3.56E-10 | 1.39E-08 |
| ENSG00000177363 | LRRN4CL | -4.099213435 | 3.64E-10 | 1.41E-08 |
| ENSG00000142185 | TRPM2 | 3.420800606 | 3.76E-10 | 1.46E-08 |
| ENSG00000224596 | ZMIZ1-AS1 | 4.223269721 | 3.84E-10 | 1.48E-08 |
| ENSG00000172578 | KLHL6 | 4.593584703 | 4.02E-10 | 1.55E-08 |
| ENSG00000127743 | IL17B | -5.73995171 | 4.28E-10 | 1.63E-08 |
| ENSG00000130066 | SAT1 | 3.02037045 | 4.32E-10 | 1.64E-08 |
| ENSG00000133805 | AMPD3 | 3.55273663 | 4.48E-10 | 1.69E-08 |
| ENSG00000133401 | PDZD2 | -3.110165948 | 4.51E-10 | 1.70E-08 |
| ENSG00000038945 | MSR1 | 4.082493763 | 4.60E-10 | 1.73E-08 |
| ENSG00000183856 | IQGAP3 | 3.903719426 | 4.73E-10 | 1.77E-08 |
| ENSG00000103742 | IGDCC4 | -3.619852247 | 4.86E-10 | 1.81E-08 |
| ENSG00000152128 | TMEM163 | 4.056407752 | 4.89E-10 | 1.82E-08 |
| ENSG00000165140 | FBP1 | 4.764076562 | 5.02E-10 | 1.87E-08 |
| ENSG00000160883 | HK3 | 3.699033057 | 5.15E-10 | 1.91E-08 |
| ENSG00000129450 | SIGLEC9 | 3.231030622 | 5.46E-10 | 2.00E-08 |
| ENSG00000133800 | LYVE1 | -3.599129788 | 5.48E-10 | 2.01E-08 |
| ENSG00000164129 | NPY5R | -3.232204084 | 5.49E-10 | 2.01E-08 |
| ENSG00000185436 | IFNLR1 | 3.58456974 | 5.59E-10 | 2.04E-08 |
| ENSG00000133962 | CATSPERB | -3.625360531 | 5.74E-10 | 2.09E-08 |
| ENSG00000076944 | STXBP2 | 3.326005116 | 5.80E-10 | 2.11E-08 |
| ENSG00000127955 | GNAI1 | -3.0977758 | 5.81E-10 | 2.11E-08 |
| ENSG00000167771 | RCOR2 | -3.652513064 | 5.94E-10 | 2.15E-08 |
| ENSG00000186462 | NAP1L2 | -3.177894889 | 6.07E-10 | 2.19E-08 |
| ENSG00000118515 | SGK1 | 3.264282691 | 6.10E-10 | 2.20E-08 |
| ENSG00000115919 | KYNU | 3.385485909 | 6.27E-10 | 2.25E-08 |
| ENSG00000118407 | FILIP1 | -3.141827262 | 6.29E-10 | 2.26E-08 |
| ENSG00000238113 | LINC01410 | 3.383357245 | 6.42E-10 | 2.30E-08 |
| ENSG00000184730 | APOBR | 3.598740261 | 6.45E-10 | 2.30E-08 |
| ENSG00000007038 | PRSS21 | 5.125970697 | 6.47E-10 | 2.30E-08 |
| ENSG00000121797 | CCRL2 | 3.809689569 | 6.89E-10 | 2.43E-08 |
| ENSG00000095970 | TREM2 | 4.230676365 | 7.00E-10 | 2.47E-08 |
| ENSG00000104081 | BMF | 3.266329929 | 7.35E-10 | 2.58E-08 |
| ENSG00000204257 | HLA-DMA | 3.187925001 | 7.36E-10 | 2.58E-08 |
| ENSG00000093072 | ADA2 | 4.070690893 | 7.39E-10 | 2.59E-08 |
| ENSG00000155629 | PIK3AP1 | 3.664321766 | 7.57E-10 | 2.64E-08 |
| ENSG00000137142 | IGFBPL1 | -3.498372772 | 7.71E-10 | 2.69E-08 |
| ENSG00000253755 | IGHGP | 4.278413917 | 7.88E-10 | 2.73E-08 |
| ENSG00000165966 | PDZRN4 | -4.022149436 | 8.30E-10 | 2.88E-08 |
| ENSG00000182103 | FAM181B | -3.42662273 | 8.38E-10 | 2.89E-08 |
| ENSG00000227066 | Z98257.1 | 5.157355045 | 8.49E-10 | 2.92E-08 |
| ENSG00000138964 | PARVG | 3.312017747 | 9.64E-10 | 3.28E-08 |
| ENSG00000104894 | CD37 | 3.712078013 | 9.67E-10 | 3.29E-08 |
| ENSG00000173432 | SAA1 | -3.539180257 | 9.80E-10 | 3.33E-08 |
| ENSG00000166148 | AVPR1A | -5.538842776 | 9.98E-10 | 3.38E-08 |
| ENSG00000117676 | RPS6KA1 | 3.671949479 | 9.99E-10 | 3.38E-08 |
| ENSG00000178789 | CD300LB | 3.646854295 | 1.02E-09 | 3.45E-08 |
| ENSG00000227502 | LINC01268 | 3.389115878 | 1.03E-09 | 3.46E-08 |
| ENSG00000122756 | CNTFR | -9.335520773 | 1.04E-09 | 3.48E-08 |
| ENSG00000234964 | FABP5P7 | 3.514303542 | 1.06E-09 | 3.54E-08 |
| ENSG00000095637 | SORBS1 | -3.098557476 | 1.07E-09 | 3.58E-08 |
| ENSG00000185046 | ANKS1B | -3.366209265 | 1.08E-09 | 3.61E-08 |
| ENSG00000254510 | AP001107.5 | -3.585476881 | 1.11E-09 | 3.71E-08 |
| ENSG00000163131 | CTSS | 4.278507763 | 1.12E-09 | 3.71E-08 |
| ENSG00000202474 | RNA5SP283 | 4.082940308 | 1.16E-09 | 3.83E-08 |
| ENSG00000175785 | PRIMA1 | -9.072432295 | 1.21E-09 | 3.98E-08 |
| ENSG00000133246 | PRAM1 | 3.40366046 | 1.28E-09 | 4.18E-08 |
| ENSG00000116701 | NCF2 | 3.490145618 | 1.33E-09 | 4.32E-08 |
| ENSG00000184811 | TRARG1 | -4.155831148 | 1.45E-09 | 4.69E-08 |
| ENSG00000102445 | RUBCNL | 3.006755298 | 1.47E-09 | 4.72E-08 |
| ENSG00000112936 | C7 | -3.343285129 | 1.48E-09 | 4.76E-08 |
| ENSG00000267123 | LINC02081 | 5.824556749 | 1.59E-09 | 5.08E-08 |
| ENSG00000147168 | IL2RG | 3.308676202 | 1.76E-09 | 5.59E-08 |
| ENSG00000211677 | IGLC2 | 3.54041631 | 1.95E-09 | 6.15E-08 |
| ENSG00000105088 | OLFM2 | -3.00019161 | 2.18E-09 | 6.78E-08 |
| ENSG00000085741 | WNT11 | -3.82647417 | 2.21E-09 | 6.88E-08 |
| ENSG00000115112 | TFCP2L1 | 4.153318481 | 2.24E-09 | 6.94E-08 |
| ENSG00000182487 | NCF1B | 3.267249516 | 2.39E-09 | 7.36E-08 |
| ENSG00000079215 | SLC1A3 | 3.307909435 | 2.40E-09 | 7.38E-08 |
| ENSG00000159399 | HK2 | 3.297469067 | 2.43E-09 | 7.46E-08 |
| ENSG00000285492 | AL356417.3 | 5.517385586 | 2.54E-09 | 7.78E-08 |
| ENSG00000235027 | AC068580.3 | 4.021967638 | 2.61E-09 | 7.95E-08 |
| ENSG00000166825 | ANPEP | 3.17365952 | 2.71E-09 | 8.21E-08 |
| ENSG00000106066 | CPVL | 3.709358882 | 2.74E-09 | 8.29E-08 |
| ENSG00000127507 | ADGRE2 | 3.616292787 | 2.98E-09 | 8.92E-08 |
| ENSG00000140678 | ITGAX | 4.282421207 | 3.09E-09 | 9.22E-08 |
| ENSG00000135077 | HAVCR2 | 3.719181636 | 3.11E-09 | 9.22E-08 |
| ENSG00000166920 | C15orf48 | 5.003493166 | 3.13E-09 | 9.28E-08 |
| ENSG00000175920 | DOK7 | -3.920860585 | 3.16E-09 | 9.37E-08 |
| ENSG00000139626 | ITGB7 | 4.080346381 | 3.50E-09 | 1.03E-07 |
| ENSG00000175352 | NRIP3 | 3.611164496 | 3.55E-09 | 1.04E-07 |
| ENSG00000180509 | KCNE1 | 3.325277895 | 3.71E-09 | 1.08E-07 |
| ENSG00000214855 | APOC1P1 | 4.198866744 | 3.86E-09 | 1.12E-07 |
| ENSG00000240405 | SAMMSON | -5.946671881 | 4.03E-09 | 1.16E-07 |
| ENSG00000211938 | IGHV3-7 | 3.694739905 | 4.10E-09 | 1.18E-07 |
| ENSG00000277089 | AC243829.4 | 4.964539491 | 4.12E-09 | 1.19E-07 |
| ENSG00000054277 | OPN3 | 3.193868675 | 4.16E-09 | 1.20E-07 |
| ENSG00000167749 | KLK4 | 8.27905564 | 4.45E-09 | 1.27E-07 |
| ENSG00000134955 | SLC37A2 | 4.139340987 | 4.46E-09 | 1.27E-07 |
| ENSG00000143119 | CD53 | 3.443183453 | 4.75E-09 | 1.34E-07 |
| ENSG00000232057 | AC093390.1 | -3.389052375 | 4.89E-09 | 1.38E-07 |
| ENSG00000165178 | NCF1C | 3.325169983 | 4.97E-09 | 1.40E-07 |
| ENSG00000147488 | ST18 | 5.019679882 | 5.00E-09 | 1.40E-07 |
| ENSG00000151117 | TMEM86A | 3.67926673 | 5.02E-09 | 1.41E-07 |
| ENSG00000177614 | PGBD5 | 4.063942376 | 5.10E-09 | 1.43E-07 |
| ENSG00000167613 | LAIR1 | 3.489407305 | 5.14E-09 | 1.43E-07 |
| ENSG00000166927 | MS4A7 | 3.417158525 | 5.24E-09 | 1.46E-07 |
| ENSG00000161921 | CXCL16 | 3.290282214 | 5.41E-09 | 1.50E-07 |
| ENSG00000108932 | SLC16A6 | 4.447462055 | 5.47E-09 | 1.51E-07 |
| ENSG00000156966 | B3GNT7 | 3.691112098 | 5.49E-09 | 1.52E-07 |
| ENSG00000160213 | CSTB | 3.594452396 | 5.58E-09 | 1.54E-07 |
| ENSG00000186074 | CD300LF | 4.371666892 | 5.75E-09 | 1.58E-07 |
| ENSG00000276231 | PIK3R6 | 3.783651615 | 6.00E-09 | 1.64E-07 |
| ENSG00000253522 | MIR3142HG | 3.946698703 | 6.14E-09 | 1.68E-07 |
| ENSG00000175567 | UCP2 | 3.192742557 | 6.35E-09 | 1.73E-07 |
| ENSG00000049249 | TNFRSF9 | 3.07427127 | 6.40E-09 | 1.74E-07 |
| ENSG00000129988 | LBP | -9.652740977 | 6.43E-09 | 1.74E-07 |
| ENSG00000250708 | LINC02269 | -6.139355417 | 6.49E-09 | 1.76E-07 |
| ENSG00000105967 | TFEC | 3.59979682 | 6.76E-09 | 1.82E-07 |
| ENSG00000121316 | PLBD1 | 3.11013932 | 6.84E-09 | 1.84E-07 |
| ENSG00000211598 | IGKV4-1 | 3.091857855 | 6.91E-09 | 1.85E-07 |
| ENSG00000169442 | CD52 | 4.398223871 | 7.11E-09 | 1.90E-07 |
| ENSG00000116254 | CHD5 | -5.188581262 | 7.51E-09 | 2.01E-07 |
| ENSG00000130203 | APOE | 4.743960615 | 7.93E-09 | 2.11E-07 |
| ENSG00000070193 | FGF10 | -9.139721331 | 8.02E-09 | 2.13E-07 |
| ENSG00000023171 | GRAMD1B | 3.023856354 | 8.28E-09 | 2.19E-07 |
| ENSG00000114013 | CD86 | 3.784494923 | 8.37E-09 | 2.21E-07 |
| ENSG00000181634 | TNFSF15 | 3.343403718 | 8.45E-09 | 2.23E-07 |
| ENSG00000278934 | AC117489.1 | -4.712649394 | 8.92E-09 | 2.33E-07 |
| ENSG00000092529 | CAPN3 | 3.139171595 | 8.97E-09 | 2.34E-07 |
| ENSG00000248290 | TNXA | -3.627992834 | 8.98E-09 | 2.34E-07 |
| ENSG00000112214 | FHL5 | -3.791784606 | 9.06E-09 | 2.36E-07 |
| ENSG00000125637 | PSD4 | 3.127624476 | 9.11E-09 | 2.37E-07 |
| ENSG00000168995 | SIGLEC7 | 3.761510928 | 9.42E-09 | 2.43E-07 |
| ENSG00000142583 | SLC2A5 | 4.622373192 | 9.43E-09 | 2.43E-07 |
| ENSG00000204632 | HLA-G | 7.367230492 | 9.54E-09 | 2.45E-07 |
| ENSG00000147642 | SYBU | -3.51875036 | 9.84E-09 | 2.51E-07 |
| ENSG00000283839 | AC096667.1 | 4.078674701 | 9.91E-09 | 2.53E-07 |
| ENSG00000183454 | GRIN2A | -3.37313999 | 1.01E-08 | 2.56E-07 |
| ENSG00000183036 | PCP4 | -8.831704231 | 1.02E-08 | 2.59E-07 |
| ENSG00000011600 | TYROBP | 3.534817575 | 1.02E-08 | 2.59E-07 |
| ENSG00000226822 | AL390036.1 | 3.52953135 | 1.15E-08 | 2.90E-07 |
| ENSG00000111012 | CYP27B1 | 5.598511935 | 1.17E-08 | 2.94E-07 |
| ENSG00000149970 | CNKSR2 | -3.227943347 | 1.18E-08 | 2.95E-07 |
| ENSG00000047597 | XK | -3.244357911 | 1.22E-08 | 3.04E-07 |
| ENSG00000154127 | UBASH3B | 3.108993931 | 1.22E-08 | 3.04E-07 |
| ENSG00000131401 | NAPSB | 3.920384831 | 1.25E-08 | 3.09E-07 |
| ENSG00000164181 | ELOVL7 | -4.019556999 | 1.25E-08 | 3.10E-07 |
| ENSG00000115705 | TPO | -4.794223816 | 1.26E-08 | 3.13E-07 |
| ENSG00000179136 | LINC00670 | -3.752963926 | 1.27E-08 | 3.14E-07 |
| ENSG00000276855 | AC015922.3 | -3.120714622 | 1.28E-08 | 3.17E-07 |
| ENSG00000196972 | SMIM10L2B | -3.492915911 | 1.35E-08 | 3.31E-07 |
| ENSG00000187808 | SOWAHD | 4.470829902 | 1.44E-08 | 3.51E-07 |
| ENSG00000070190 | DAPP1 | 3.55097098 | 1.46E-08 | 3.56E-07 |
| ENSG00000165197 | VEGFD | -3.797359287 | 1.49E-08 | 3.62E-07 |
| ENSG00000160255 | ITGB2 | 3.683834255 | 1.55E-08 | 3.75E-07 |
| ENSG00000186451 | SPATA12 | 4.957117895 | 1.59E-08 | 3.84E-07 |
| ENSG00000226124 | FTCDNL1 | 3.416936119 | 1.63E-08 | 3.91E-07 |
| ENSG00000188488 | SERPINA5 | -5.370264293 | 1.68E-08 | 4.02E-07 |
| ENSG00000163814 | CDCP1 | 3.454126646 | 1.72E-08 | 4.12E-07 |
| ENSG00000254851 | AP005018.2 | -3.754022493 | 1.85E-08 | 4.40E-07 |
| ENSG00000104044 | OCA2 | -4.626524568 | 1.92E-08 | 4.56E-07 |
| ENSG00000157766 | ACAN | -3.641763652 | 1.93E-08 | 4.57E-07 |
| ENSG00000134020 | PEBP4 | -3.905166171 | 1.93E-08 | 4.57E-07 |
| ENSG00000203747 | FCGR3A | 3.423397605 | 1.99E-08 | 4.69E-07 |
| ENSG00000092621 | PHGDH | -3.75022092 | 1.99E-08 | 4.69E-07 |
| ENSG00000126882 | FAM78A | 3.142697269 | 2.31E-08 | 5.37E-07 |
| ENSG00000182566 | CLEC4G | -3.301069758 | 2.33E-08 | 5.40E-07 |
| ENSG00000171551 | ECEL1 | -6.865809434 | 2.45E-08 | 5.66E-07 |
| ENSG00000244437 | IGKV3-15 | 3.600680401 | 2.51E-08 | 5.77E-07 |
| ENSG00000069424 | KCNAB2 | 3.33797978 | 2.51E-08 | 5.77E-07 |
| ENSG00000172724 | CCL19 | -3.424207365 | 2.77E-08 | 6.30E-07 |
| ENSG00000114646 | CSPG5 | -4.025038445 | 2.79E-08 | 6.35E-07 |
| ENSG00000104369 | JPH1 | -5.525639124 | 2.84E-08 | 6.46E-07 |
| ENSG00000197635 | DPP4 | 4.395897496 | 2.92E-08 | 6.60E-07 |
| ENSG00000162989 | KCNJ3 | -4.05846533 | 3.02E-08 | 6.81E-07 |
| ENSG00000235385 | LINC02154 | 4.731473705 | 3.04E-08 | 6.83E-07 |
| ENSG00000135549 | PKIB | 3.694511329 | 3.10E-08 | 6.95E-07 |
| ENSG00000136167 | LCP1 | 3.192282885 | 3.30E-08 | 7.39E-07 |
| ENSG00000211897 | IGHG3 | 4.005996858 | 3.37E-08 | 7.52E-07 |
| ENSG00000087086 | FTL | 3.081500484 | 3.38E-08 | 7.53E-07 |
| ENSG00000185437 | SH3BGR | -3.110307261 | 3.44E-08 | 7.66E-07 |
| ENSG00000102760 | RGCC | 3.220608534 | 3.47E-08 | 7.72E-07 |
| ENSG00000169302 | STK32A | -3.027670218 | 3.48E-08 | 7.73E-07 |
| ENSG00000185313 | SCN10A | 4.733465315 | 3.70E-08 | 8.15E-07 |
| ENSG00000167208 | SNX20 | 3.558926124 | 3.75E-08 | 8.23E-07 |
| ENSG00000029559 | IBSP | 9.642938348 | 3.97E-08 | 8.64E-07 |
| ENSG00000146411 | SLC2A12 | -3.18629266 | 3.99E-08 | 8.67E-07 |
| ENSG00000025434 | NR1H3 | 3.381692933 | 3.99E-08 | 8.67E-07 |
| ENSG00000233384 | AC096537.1 | 8.371233961 | 4.05E-08 | 8.79E-07 |
| ENSG00000012779 | ALOX5 | 3.447991004 | 4.06E-08 | 8.81E-07 |
| ENSG00000136546 | SCN7A | -8.998958813 | 4.12E-08 | 8.93E-07 |
| ENSG00000104267 | CA2 | 4.453247399 | 4.16E-08 | 9.00E-07 |
| ENSG00000253490 | LINC02099 | 7.477489201 | 4.31E-08 | 9.27E-07 |
| ENSG00000178015 | GPR150 | 3.797960571 | 4.52E-08 | 9.66E-07 |
| ENSG00000204882 | GPR20 | -4.421808565 | 4.64E-08 | 9.88E-07 |
| ENSG00000168772 | CXXC4 | -4.946316764 | 4.72E-08 | 1.00E-06 |
| ENSG00000235568 | NFAM1 | 3.665142838 | 5.15E-08 | 1.09E-06 |
| ENSG00000134242 | PTPN22 | 3.230937577 | 5.23E-08 | 1.11E-06 |
| ENSG00000183019 | MCEMP1 | 4.137787346 | 5.47E-08 | 1.15E-06 |
| ENSG00000130595 | TNNT3 | -3.439472993 | 5.77E-08 | 1.21E-06 |
| ENSG00000100985 | MMP9 | 9.283161377 | 6.11E-08 | 1.28E-06 |
| ENSG00000019582 | CD74 | 3.238769673 | 6.18E-08 | 1.29E-06 |
| ENSG00000180875 | GREM2 | -6.95651195 | 6.48E-08 | 1.34E-06 |
| ENSG00000142512 | SIGLEC10 | 3.438561019 | 6.91E-08 | 1.42E-06 |
| ENSG00000223552 | AC098613.1 | 3.392036516 | 6.93E-08 | 1.43E-06 |
| ENSG00000279406 | AL359183.1 | 4.340535843 | 7.24E-08 | 1.48E-06 |
| ENSG00000259590 | LINC02244 | 5.133548264 | 7.37E-08 | 1.51E-06 |
| ENSG00000234438 | KBTBD13 | -8.981189209 | 7.49E-08 | 1.53E-06 |
| ENSG00000108381 | ASPA | -5.269054523 | 7.62E-08 | 1.55E-06 |
| ENSG00000050030 | NEXMIF | -3.29494433 | 8.12E-08 | 1.64E-06 |
| ENSG00000141506 | PIK3R5 | 3.286644494 | 8.17E-08 | 1.65E-06 |
| ENSG00000141968 | VAV1 | 3.001280837 | 8.44E-08 | 1.70E-06 |
| ENSG00000086300 | SNX10 | 3.82724297 | 8.50E-08 | 1.71E-06 |
| ENSG00000203446 | AC004988.1 | 4.308132274 | 8.60E-08 | 1.73E-06 |
| ENSG00000110079 | MS4A4A | 3.505430741 | 8.72E-08 | 1.75E-06 |
| ENSG00000124440 | HIF3A | -3.214123989 | 8.74E-08 | 1.75E-06 |
| ENSG00000164879 | CA3 | -8.362396955 | 8.78E-08 | 1.75E-06 |
| ENSG00000140600 | SH3GL3 | -8.496020929 | 9.10E-08 | 1.81E-06 |
| ENSG00000040608 | RTN4R | 3.280519723 | 9.11E-08 | 1.81E-06 |
| ENSG00000112218 | GPR63 | -3.34576004 | 9.19E-08 | 1.83E-06 |
| ENSG00000138615 | CILP | -3.922174364 | 9.26E-08 | 1.84E-06 |
| ENSG00000128604 | IRF5 | 3.134764323 | 9.56E-08 | 1.89E-06 |
| ENSG00000118557 | PMFBP1 | 3.944585821 | 9.69E-08 | 1.91E-06 |
| ENSG00000204060 | FOXO6 | -3.409685341 | 9.70E-08 | 1.91E-06 |
| ENSG00000137101 | CD72 | 3.445519991 | 9.88E-08 | 1.94E-06 |
| ENSG00000113532 | ST8SIA4 | 3.019151048 | 9.94E-08 | 1.95E-06 |
| ENSG00000217801 | AL390719.1 | 4.349591573 | 1.07E-07 | 2.09E-06 |
| ENSG00000152315 | KCNK13 | 3.646324821 | 1.08E-07 | 2.11E-06 |
| ENSG00000162777 | DENND2D | 3.146191102 | 1.15E-07 | 2.23E-06 |
| ENSG00000174130 | TLR6 | 3.123799167 | 1.23E-07 | 2.37E-06 |
| ENSG00000135678 | CPM | 3.978672262 | 1.23E-07 | 2.38E-06 |
| ENSG00000285768 | AC232323.2 | -5.363251404 | 1.26E-07 | 2.43E-06 |
| ENSG00000203930 | LINC00632 | -3.350021712 | 1.27E-07 | 2.45E-06 |
| ENSG00000163563 | MNDA | 3.436004381 | 1.34E-07 | 2.55E-06 |
| ENSG00000134061 | CD180 | 3.691748178 | 1.34E-07 | 2.55E-06 |
| ENSG00000147647 | DPYS | 3.979547035 | 1.39E-07 | 2.62E-06 |
| ENSG00000122824 | NUDT10 | -4.99323427 | 1.44E-07 | 2.72E-06 |
| ENSG00000204252 | HLA-DOA | 3.431849549 | 1.49E-07 | 2.79E-06 |
| ENSG00000243055 | GK-AS1 | 5.258230963 | 1.51E-07 | 2.83E-06 |
| ENSG00000136040 | PLXNC1 | 3.4060184 | 1.66E-07 | 3.08E-06 |
| ENSG00000157087 | ATP2B2 | 3.87819113 | 1.69E-07 | 3.13E-06 |
| ENSG00000112303 | VNN2 | 3.422290128 | 1.71E-07 | 3.15E-06 |
| ENSG00000185565 | LSAMP | -6.013242814 | 1.73E-07 | 3.18E-06 |
| ENSG00000205683 | DPF3 | -3.231740033 | 1.73E-07 | 3.19E-06 |
| ENSG00000267405 | AC005180.1 | -3.167780948 | 1.79E-07 | 3.28E-06 |
| ENSG00000107798 | LIPA | 3.422921959 | 1.79E-07 | 3.28E-06 |
| ENSG00000062038 | CDH3 | 4.539701514 | 1.80E-07 | 3.28E-06 |
| ENSG00000069011 | PITX1 | -4.975067444 | 1.84E-07 | 3.35E-06 |
| ENSG00000241886 | AC112496.1 | 5.587127022 | 1.90E-07 | 3.46E-06 |
| ENSG00000167600 | CYP2S1 | 3.233674619 | 1.91E-07 | 3.47E-06 |
| ENSG00000101336 | HCK | 3.187141237 | 1.97E-07 | 3.58E-06 |
| ENSG00000120280 | CXorf21 | 3.701505989 | 1.98E-07 | 3.59E-06 |
| ENSG00000158517 | NCF1 | 3.346017016 | 2.02E-07 | 3.64E-06 |
| ENSG00000160791 | CCR5 | 3.155542953 | 2.06E-07 | 3.70E-06 |
| ENSG00000164695 | CHMP4C | -3.20270167 | 2.11E-07 | 3.79E-06 |
| ENSG00000248144 | ADH1C | -5.99795409 | 2.15E-07 | 3.85E-06 |
| ENSG00000203685 | STUM | -3.325366275 | 2.18E-07 | 3.90E-06 |
| ENSG00000099864 | PALM | -3.321892931 | 2.28E-07 | 4.06E-06 |
| ENSG00000206077 | ZDHHC11B | -3.414810953 | 2.30E-07 | 4.09E-06 |
| ENSG00000094796 | KRT31 | 4.641487189 | 2.30E-07 | 4.09E-06 |
| ENSG00000196376 | SLC35F1 | -5.394337096 | 2.35E-07 | 4.18E-06 |
| ENSG00000183873 | SCN5A | -3.430558476 | 2.57E-07 | 4.54E-06 |
| ENSG00000176177 | ENTHD1 | 7.756250711 | 2.68E-07 | 4.72E-06 |
| ENSG00000137841 | PLCB2 | 3.093507775 | 2.85E-07 | 4.98E-06 |
| ENSG00000100167 | SEPT3 | 3.533192094 | 2.86E-07 | 4.99E-06 |
| ENSG00000211679 | IGLC3 | 3.376203986 | 2.86E-07 | 4.99E-06 |
| ENSG00000234232 | AC243772.3 | 3.169417861 | 2.91E-07 | 5.07E-06 |
| ENSG00000272235 | AL590438.1 | -4.395156703 | 2.95E-07 | 5.12E-06 |
| ENSG00000117091 | CD48 | 3.515195255 | 2.97E-07 | 5.15E-06 |
| ENSG00000004776 | HSPB6 | -3.080412754 | 3.03E-07 | 5.25E-06 |
| ENSG00000182771 | GRID1 | -3.253876859 | 3.04E-07 | 5.25E-06 |
| ENSG00000118432 | CNR1 | -3.234970489 | 3.22E-07 | 5.54E-06 |
| ENSG00000121871 | SLITRK3 | -5.949163144 | 3.30E-07 | 5.66E-06 |
| ENSG00000180113 | TDRD6 | 3.187833754 | 3.30E-07 | 5.67E-06 |
| ENSG00000196932 | TMEM26 | 3.33741552 | 3.40E-07 | 5.83E-06 |
| ENSG00000184515 | BEX5 | -3.364907941 | 3.42E-07 | 5.86E-06 |
| ENSG00000080854 | IGSF9B | -3.353847962 | 3.52E-07 | 6.01E-06 |
| ENSG00000282572 | AC215522.2 | 4.5427284 | 3.58E-07 | 6.10E-06 |
| ENSG00000211935 | IGHV1-3 | 4.663986808 | 3.58E-07 | 6.10E-06 |
| ENSG00000173559 | NABP1 | 3.070634476 | 3.59E-07 | 6.10E-06 |
| ENSG00000274286 | ADRA2B | -3.740971832 | 3.59E-07 | 6.10E-06 |
| ENSG00000168389 | MFSD2A | 3.261165887 | 3.73E-07 | 6.30E-06 |
| ENSG00000108924 | HLF | -3.40281209 | 3.75E-07 | 6.32E-06 |
| ENSG00000056998 | GYG2 | -3.422406327 | 3.77E-07 | 6.35E-06 |
| ENSG00000211642 | IGLV10-54 | 7.703073066 | 3.89E-07 | 6.55E-06 |
| ENSG00000213876 | RPL7AP64 | 4.34206219 | 3.97E-07 | 6.65E-06 |
| ENSG00000166924 | NYAP1 | -4.005638164 | 4.06E-07 | 6.77E-06 |
| ENSG00000141505 | ASGR1 | 3.074603497 | 4.16E-07 | 6.90E-06 |
| ENSG00000196666 | FAM180B | -5.703534019 | 4.19E-07 | 6.94E-06 |
| ENSG00000007264 | MATK | 3.783778378 | 4.26E-07 | 7.05E-06 |
| ENSG00000163376 | KBTBD8 | 3.298847042 | 4.36E-07 | 7.21E-06 |
| ENSG00000163694 | RBM47 | 3.084085971 | 4.47E-07 | 7.36E-06 |
| ENSG00000175874 | CREG2 | 7.503828396 | 4.57E-07 | 7.51E-06 |
| ENSG00000069431 | ABCC9 | -3.614848118 | 4.58E-07 | 7.52E-06 |
| ENSG00000133454 | MYO18B | -3.756777924 | 4.68E-07 | 7.67E-06 |
| ENSG00000134762 | DSC3 | -8.140734743 | 4.78E-07 | 7.81E-06 |
| ENSG00000108379 | WNT3 | -3.490741514 | 4.79E-07 | 7.81E-06 |
| ENSG00000143507 | DUSP10 | 3.023297551 | 4.79E-07 | 7.82E-06 |
| ENSG00000178878 | APOLD1 | -3.302852148 | 4.84E-07 | 7.87E-06 |
| ENSG00000167588 | GPD1 | -4.353123951 | 4.86E-07 | 7.90E-06 |
| ENSG00000082074 | FYB1 | 3.380820453 | 5.06E-07 | 8.17E-06 |
| ENSG00000267060 | PTGES3L | -5.795214502 | 5.12E-07 | 8.25E-06 |
| ENSG00000122122 | SASH3 | 3.024100925 | 5.19E-07 | 8.36E-06 |
| ENSG00000185862 | EVI2B | 3.334617836 | 5.38E-07 | 8.62E-06 |
| ENSG00000273796 | BX322562.1 | -4.416283984 | 5.54E-07 | 8.86E-06 |
| ENSG00000196353 | CPNE4 | -4.926138461 | 5.82E-07 | 9.28E-06 |
| ENSG00000105825 | TFPI2 | -3.066049688 | 5.85E-07 | 9.31E-06 |
| ENSG00000110876 | SELPLG | 3.415096793 | 5.94E-07 | 9.44E-06 |
| ENSG00000112818 | MEP1A | 7.158967514 | 6.02E-07 | 9.54E-06 |
| ENSG00000140968 | IRF8 | 3.278358816 | 6.10E-07 | 9.65E-06 |
| ENSG00000163735 | CXCL5 | 5.219611167 | 6.12E-07 | 9.68E-06 |
| ENSG00000141449 | GREB1L | -3.29623574 | 6.13E-07 | 9.69E-06 |
| ENSG00000128578 | STRIP2 | 4.001958596 | 6.27E-07 | 9.87E-06 |
| ENSG00000169403 | PTAFR | 3.442492548 | 6.42E-07 | 1.01E-05 |
| ENSG00000152213 | ARL11 | 3.35700309 | 6.59E-07 | 1.03E-05 |
| ENSG00000147872 | PLIN2 | 3.074312739 | 6.60E-07 | 1.03E-05 |
| ENSG00000230006 | ANKRD36BP2 | 3.697715367 | 6.80E-07 | 1.06E-05 |
| ENSG00000121895 | TMEM156 | 3.254289863 | 6.84E-07 | 1.07E-05 |
| ENSG00000126860 | EVI2A | 3.016844888 | 6.92E-07 | 1.08E-05 |
| ENSG00000276289 | KCNE1B | 4.862298832 | 7.02E-07 | 1.09E-05 |
| ENSG00000211896 | IGHG1 | 3.130616962 | 7.04E-07 | 1.09E-05 |
| ENSG00000231389 | HLA-DPA1 | 3.563395904 | 7.14E-07 | 1.11E-05 |
| ENSG00000072133 | RPS6KA6 | -3.689991576 | 7.17E-07 | 1.11E-05 |
| ENSG00000101349 | PAK5 | -7.883604052 | 7.18E-07 | 1.11E-05 |
| ENSG00000107736 | CDH23 | 3.266424208 | 7.41E-07 | 1.14E-05 |
| ENSG00000039537 | C6 | -7.78841235 | 7.78E-07 | 1.20E-05 |
| ENSG00000010671 | BTK | 3.242524012 | 7.94E-07 | 1.22E-05 |
| ENSG00000204071 | TCEAL6 | -7.474029791 | 7.96E-07 | 1.22E-05 |
| ENSG00000142405 | NLRP12 | 3.694240846 | 8.13E-07 | 1.24E-05 |
| ENSG00000089041 | P2RX7 | 3.23027205 | 8.23E-07 | 1.26E-05 |
| ENSG00000211592 | IGKC | 3.172919846 | 8.39E-07 | 1.28E-05 |
| ENSG00000158869 | FCER1G | 3.2249327 | 8.58E-07 | 1.30E-05 |
| ENSG00000162543 | UBXN10 | 3.374334952 | 8.65E-07 | 1.31E-05 |
| ENSG00000079931 | MOXD1 | 3.12654613 | 8.65E-07 | 1.31E-05 |
| ENSG00000175793 | SFN | -6.137215429 | 8.99E-07 | 1.36E-05 |
| ENSG00000242574 | HLA-DMB | 3.456724522 | 9.02E-07 | 1.37E-05 |
| ENSG00000137766 | UNC13C | -3.394063432 | 9.29E-07 | 1.40E-05 |
| ENSG00000198829 | SUCNR1 | 3.100316217 | 9.49E-07 | 1.43E-05 |
| ENSG00000174945 | AMZ1 | 3.9500145 | 9.72E-07 | 1.46E-05 |
| ENSG00000197576 | HOXA4 | -3.542836099 | 9.87E-07 | 1.48E-05 |
| ENSG00000179915 | NRXN1 | -4.865653682 | 9.90E-07 | 1.49E-05 |
| ENSG00000274508 | AC007336.2 | 6.932157933 | 9.90E-07 | 1.49E-05 |
| ENSG00000106785 | TRIM14 | 3.030240014 | 9.98E-07 | 1.50E-05 |
| ENSG00000134516 | DOCK2 | 3.273326073 | 1.01E-06 | 1.50E-05 |
| ENSG00000165238 | WNK2 | -5.617040314 | 1.01E-06 | 1.51E-05 |
| ENSG00000137868 | STRA6 | 3.457485189 | 1.01E-06 | 1.51E-05 |
| ENSG00000168004 | HRASLS5 | -4.079439578 | 1.02E-06 | 1.52E-05 |
| ENSG00000188517 | COL25A1 | -3.785069195 | 1.05E-06 | 1.56E-05 |
| ENSG00000102290 | PCDH11X | -4.865746773 | 1.07E-06 | 1.58E-05 |
| ENSG00000050555 | LAMC3 | -3.167159952 | 1.07E-06 | 1.58E-05 |
| ENSG00000134121 | CHL1 | -7.941326467 | 1.07E-06 | 1.59E-05 |
| ENSG00000259869 | AL022344.2 | -3.957399883 | 1.08E-06 | 1.60E-05 |
| ENSG00000255398 | HCAR3 | 3.765068745 | 1.12E-06 | 1.65E-05 |
| ENSG00000163568 | AIM2 | 3.180018339 | 1.17E-06 | 1.72E-05 |
| ENSG00000077274 | CAPN6 | -5.93214537 | 1.17E-06 | 1.72E-05 |
| ENSG00000187288 | CIDEC | -7.029129238 | 1.21E-06 | 1.77E-05 |
| ENSG00000162849 | KIF26B | 3.054498959 | 1.24E-06 | 1.81E-05 |
| ENSG00000100473 | COCH | -3.516960573 | 1.25E-06 | 1.83E-05 |
| ENSG00000130598 | TNNI2 | 3.066482703 | 1.29E-06 | 1.88E-05 |
| ENSG00000103888 | CEMIP | 3.60397402 | 1.33E-06 | 1.93E-05 |
| ENSG00000187957 | DNER | -5.742958695 | 1.37E-06 | 1.97E-05 |
| ENSG00000119535 | CSF3R | 3.326866976 | 1.41E-06 | 2.03E-05 |
| ENSG00000225370 | AC243962.1 | 4.643169698 | 1.41E-06 | 2.03E-05 |
| ENSG00000186152 | LILRP1 | 7.983676786 | 1.48E-06 | 2.12E-05 |
| ENSG00000115956 | PLEK | 3.271516735 | 1.48E-06 | 2.12E-05 |
| ENSG00000176049 | JAKMIP2 | 4.324901376 | 1.54E-06 | 2.20E-05 |
| ENSG00000134201 | GSTM5 | -3.074074495 | 1.54E-06 | 2.20E-05 |
| ENSG00000204335 | SP5 | -8.297542551 | 1.56E-06 | 2.22E-05 |
| ENSG00000224940 | PRRT4 | -6.293704308 | 1.57E-06 | 2.23E-05 |
| ENSG00000133048 | CHI3L1 | 6.316562648 | 1.58E-06 | 2.24E-05 |
| ENSG00000064547 | LPAR2 | 3.007440559 | 1.59E-06 | 2.25E-05 |
| ENSG00000232079 | LINC01697 | -6.985971643 | 1.60E-06 | 2.26E-05 |
| ENSG00000164107 | HAND2 | -4.043603557 | 1.61E-06 | 2.28E-05 |
| ENSG00000065609 | SNAP91 | -3.834086623 | 1.65E-06 | 2.33E-05 |
| ENSG00000273812 | BX640514.2 | 3.464052686 | 1.70E-06 | 2.39E-05 |
| ENSG00000137491 | SLCO2B1 | 3.144162359 | 1.72E-06 | 2.41E-05 |
| ENSG00000069482 | GAL | 3.842753904 | 1.72E-06 | 2.41E-05 |
| ENSG00000151365 | THRSP | -10.93279457 | 1.72E-06 | 2.41E-05 |
| ENSG00000251230 | MIR3945HG | 3.950934832 | 1.73E-06 | 2.42E-05 |
| ENSG00000166923 | GREM1 | 3.814727827 | 1.91E-06 | 2.65E-05 |
| ENSG00000100276 | RASL10A | 3.270255705 | 1.96E-06 | 2.71E-05 |
| ENSG00000187474 | FPR3 | 3.271328523 | 1.97E-06 | 2.72E-05 |
| ENSG00000139767 | SRRM4 | -5.696036371 | 1.98E-06 | 2.74E-05 |
| ENSG00000186517 | ARHGAP30 | 3.062830336 | 1.99E-06 | 2.75E-05 |
| ENSG00000203710 | CR1 | 3.840165316 | 2.04E-06 | 2.80E-05 |
| ENSG00000162723 | SLAMF9 | 7.67338842 | 2.07E-06 | 2.83E-05 |
| ENSG00000231964 | AL731567.1 | 3.655369362 | 2.07E-06 | 2.84E-05 |
| ENSG00000147588 | PMP2 | -8.515598024 | 2.08E-06 | 2.84E-05 |
| ENSG00000280087 | AC011481.3 | 3.104343792 | 2.10E-06 | 2.87E-05 |
| ENSG00000170775 | GPR37 | -3.228557398 | 2.11E-06 | 2.88E-05 |
| ENSG00000282608 | ADORA3 | 3.329289721 | 2.20E-06 | 2.99E-05 |
| ENSG00000071991 | CDH19 | -3.640062176 | 2.25E-06 | 3.06E-05 |
| ENSG00000181778 | TMEM252 | -3.329771553 | 2.39E-06 | 3.22E-05 |
| ENSG00000223534 | HLA-DQB1-AS1 | 4.487627831 | 2.54E-06 | 3.42E-05 |
| ENSG00000135472 | FAIM2 | -3.732329839 | 2.59E-06 | 3.48E-05 |
| ENSG00000188613 | NANOS1 | -3.167869571 | 2.70E-06 | 3.62E-05 |
| ENSG00000253958 | CLDN23 | 3.070570888 | 2.81E-06 | 3.76E-05 |
| ENSG00000196517 | SLC6A9 | -3.301033603 | 2.83E-06 | 3.77E-05 |
| ENSG00000228035 | NGF-AS1 | -4.128993776 | 2.93E-06 | 3.90E-05 |
| ENSG00000143297 | FCRL5 | 3.344226597 | 2.94E-06 | 3.91E-05 |
| ENSG00000279204 | AC134043.2 | -3.738763435 | 2.96E-06 | 3.93E-05 |
| ENSG00000231131 | LNCAROD | 4.849880408 | 2.97E-06 | 3.95E-05 |
| ENSG00000204287 | HLA-DRA | 3.320181553 | 3.01E-06 | 3.99E-05 |
| ENSG00000164841 | TMEM74 | -5.483901932 | 3.08E-06 | 4.08E-05 |
| ENSG00000123338 | NCKAP1L | 3.137389437 | 3.16E-06 | 4.17E-05 |
| ENSG00000126337 | KRT36 | 6.914299617 | 3.16E-06 | 4.17E-05 |
| ENSG00000167261 | DPEP2 | 3.849659587 | 3.29E-06 | 4.33E-05 |
| ENSG00000114200 | BCHE | -3.684696212 | 3.29E-06 | 4.33E-05 |
| ENSG00000279484 | KLHL30-AS1 | -4.157125389 | 3.32E-06 | 4.36E-05 |
| ENSG00000145934 | TENM2 | -6.208732823 | 3.34E-06 | 4.38E-05 |
| ENSG00000235897 | TM4SF19-AS1 | 4.833525403 | 3.37E-06 | 4.41E-05 |
| ENSG00000266835 | GAPLINC | 3.574468791 | 3.38E-06 | 4.42E-05 |
| ENSG00000146678 | IGFBP1 | 6.163921135 | 3.52E-06 | 4.59E-05 |
| ENSG00000182782 | HCAR2 | 3.169421351 | 3.59E-06 | 4.67E-05 |
| ENSG00000258498 | DIO3OS | -6.971176183 | 3.72E-06 | 4.83E-05 |
| ENSG00000261713 | SSTR5-AS1 | -7.888089437 | 3.85E-06 | 4.96E-05 |
| ENSG00000233834 | AC005083.1 | 3.239621916 | 3.98E-06 | 5.12E-05 |
| ENSG00000176956 | LY6H | 5.289061476 | 4.01E-06 | 5.14E-05 |
| ENSG00000174938 | SEZ6L2 | 3.372208219 | 4.08E-06 | 5.23E-05 |
| ENSG00000270980 | AC060764.1 | 6.8543546 | 4.17E-06 | 5.33E-05 |
| ENSG00000154736 | ADAMTS5 | -3.011997168 | 4.37E-06 | 5.56E-05 |
| ENSG00000102385 | DRP2 | -5.008825453 | 4.40E-06 | 5.59E-05 |
| ENSG00000117115 | PADI2 | 4.024141589 | 4.48E-06 | 5.69E-05 |
| ENSG00000211670 | IGLV3-9 | 4.088668547 | 4.52E-06 | 5.72E-05 |
| ENSG00000173376 | NDNF | -4.284217464 | 4.60E-06 | 5.82E-05 |
| ENSG00000169427 | KCNK9 | -4.384672674 | 4.68E-06 | 5.91E-05 |
| ENSG00000260428 | SCX | -3.599879107 | 4.71E-06 | 5.95E-05 |
| ENSG00000155719 | OTOA | 3.756232013 | 4.80E-06 | 6.05E-05 |
| ENSG00000185477 | GPRIN3 | 3.279982628 | 4.82E-06 | 6.07E-05 |
| ENSG00000125538 | IL1B | 3.397235269 | 4.97E-06 | 6.24E-05 |
| ENSG00000171864 | PRND | 7.365382275 | 5.03E-06 | 6.32E-05 |
| ENSG00000078549 | ADCYAP1R1 | -6.70750471 | 5.04E-06 | 6.32E-05 |
| ENSG00000126890 | CTAG2 | -20.12231043 | 5.04E-06 | 6.32E-05 |
| ENSG00000152977 | ZIC1 | -20.09244222 | 5.19E-06 | 6.48E-05 |
| ENSG00000010610 | CD4 | 3.367793903 | 5.23E-06 | 6.52E-05 |
| ENSG00000241755 | IGKV1-9 | 5.105820435 | 5.43E-06 | 6.74E-05 |
| ENSG00000198597 | ZNF536 | -7.578323579 | 5.56E-06 | 6.89E-05 |
| ENSG00000100884 | CPNE6 | -8.235048598 | 5.56E-06 | 6.89E-05 |
| ENSG00000234191 | AC091808.1 | 6.5694239 | 5.98E-06 | 7.36E-05 |
| ENSG00000107165 | TYRP1 | -3.300006265 | 6.32E-06 | 7.75E-05 |
| ENSG00000145703 | IQGAP2 | 3.224637226 | 6.49E-06 | 7.95E-05 |
| ENSG00000175093 | SPSB4 | -4.813999164 | 6.57E-06 | 8.03E-05 |
| ENSG00000179331 | RAB39A | 3.24103051 | 6.60E-06 | 8.06E-05 |
| ENSG00000069812 | HES2 | 6.374639204 | 6.88E-06 | 8.37E-05 |
| ENSG00000241935 | HOGA1 | -3.699839056 | 6.89E-06 | 8.38E-05 |
| ENSG00000163121 | NEURL3 | 3.032550789 | 6.90E-06 | 8.39E-05 |
| ENSG00000143226 | FCGR2A | 3.00010288 | 6.94E-06 | 8.43E-05 |
| ENSG00000100146 | SOX10 | -5.185884637 | 7.15E-06 | 8.67E-05 |
| ENSG00000119866 | BCL11A | 4.332964967 | 7.21E-06 | 8.73E-05 |
| ENSG00000059377 | TBXAS1 | 3.164554087 | 7.26E-06 | 8.79E-05 |
| ENSG00000134460 | IL2RA | 3.317870027 | 7.33E-06 | 8.87E-05 |
| ENSG00000218336 | TENM3 | -4.764439462 | 7.56E-06 | 9.12E-05 |
| ENSG00000251493 | FOXD1 | -3.695736564 | 7.95E-06 | 9.54E-05 |
| ENSG00000276980 | AC008760.2 | -3.327717732 | 8.23E-06 | 9.81E-05 |
| ENSG00000232679 | LINC01705 | 7.021840074 | 8.90E-06 | 0.000105623 |
| ENSG00000185527 | PDE6G | 4.432093172 | 9.19E-06 | 0.000108721 |
| ENSG00000211668 | IGLV2-11 | 3.288923707 | 9.36E-06 | 0.000110351 |
| ENSG00000165300 | SLITRK5 | -4.023663432 | 9.40E-06 | 0.000110752 |
| ENSG00000132821 | VSTM2L | -4.554625244 | 9.46E-06 | 0.000111321 |
| ENSG00000183571 | PGPEP1L | -7.222681289 | 9.58E-06 | 0.000112599 |
| ENSG00000261596 | AC005632.2 | 3.20055713 | 9.63E-06 | 0.000113099 |
| ENSG00000104833 | TUBB4A | 3.492073961 | 9.84E-06 | 0.000115258 |
| ENSG00000241158 | ADAMTS9-AS1 | -5.559950412 | 9.89E-06 | 0.000115749 |
| ENSG00000163347 | CLDN1 | 3.657436287 | 1.01E-05 | 0.000117902 |
| ENSG00000231419 | LINC00689 | -3.895922963 | 1.04E-05 | 0.00012098 |
| ENSG00000165685 | TMEM52B | 3.730695414 | 1.04E-05 | 0.00012098 |
| ENSG00000211947 | IGHV3-21 | 3.754664182 | 1.05E-05 | 0.000121914 |
| ENSG00000236333 | TRHDE-AS1 | -3.048216242 | 1.06E-05 | 0.000122554 |
| ENSG00000120907 | ADRA1A | -8.054086134 | 1.06E-05 | 0.000123467 |
| ENSG00000162551 | ALPL | -3.07864957 | 1.08E-05 | 0.00012456 |
| ENSG00000161911 | TREML1 | 3.064032551 | 1.08E-05 | 0.000124754 |
| ENSG00000101916 | TLR8 | 3.215403382 | 1.10E-05 | 0.000126894 |
| ENSG00000165023 | DIRAS2 | 5.097958524 | 1.15E-05 | 0.000132756 |
| ENSG00000250929 | LINC01181 | 7.398990616 | 1.17E-05 | 0.000133901 |
| ENSG00000250155 | AC008957.1 | 3.955381727 | 1.17E-05 | 0.000134608 |
| ENSG00000147434 | CHRNA6 | 7.19538855 | 1.19E-05 | 0.000135885 |
| ENSG00000196220 | SRGAP3 | 3.45578797 | 1.31E-05 | 0.000149043 |
| ENSG00000136943 | CTSV | 3.424126208 | 1.35E-05 | 0.000152801 |
| ENSG00000162009 | SSTR5 | -4.446792603 | 1.37E-05 | 0.000154605 |
| ENSG00000255921 | AC026310.2 | 6.740109959 | 1.40E-05 | 0.000157085 |
| ENSG00000250986 | LINC02600 | -6.385899669 | 1.40E-05 | 0.000157737 |
| ENSG00000134755 | DSC2 | 3.338024799 | 1.42E-05 | 0.000159562 |
| ENSG00000149575 | SCN2B | -6.45010328 | 1.44E-05 | 0.000161392 |
| ENSG00000140479 | PCSK6 | 3.309617689 | 1.45E-05 | 0.000162229 |
| ENSG00000160951 | PTGER1 | -3.542994226 | 1.53E-05 | 0.000171099 |
| ENSG00000274029 | AC069209.1 | 3.952190673 | 1.54E-05 | 0.00017113 |
| ENSG00000167419 | LPO | 5.417502974 | 1.58E-05 | 0.000175462 |
| ENSG00000165449 | SLC16A9 | -3.332748195 | 1.58E-05 | 0.000175469 |
| ENSG00000167748 | KLK1 | 5.734932445 | 1.63E-05 | 0.000180422 |
| ENSG00000273295 | AP000350.6 | -7.561578299 | 1.64E-05 | 0.000180747 |
| ENSG00000274248 | AJ011932.1 | -3.792776312 | 1.65E-05 | 0.000182529 |
| ENSG00000172901 | LVRN | -3.767449152 | 1.68E-05 | 0.000184735 |
| ENSG00000158714 | SLAMF8 | 3.73399418 | 1.72E-05 | 0.000189097 |
| ENSG00000197561 | ELANE | -6.658587177 | 1.75E-05 | 0.000192537 |
| ENSG00000211943 | IGHV3-15 | 3.284351747 | 1.77E-05 | 0.000194299 |
| ENSG00000165025 | SYK | 3.411097 | 1.78E-05 | 0.000194787 |
| ENSG00000101440 | ASIP | 5.196400959 | 1.79E-05 | 0.000195873 |
| ENSG00000149295 | DRD2 | -4.97196277 | 1.79E-05 | 0.000196026 |
| ENSG00000258791 | LINC00520 | 5.835834493 | 1.82E-05 | 0.000199099 |
| ENSG00000241294 | IGKV2-24 | 3.044255732 | 1.83E-05 | 0.000199491 |
| ENSG00000257838 | OTOAP1 | 4.119352571 | 1.96E-05 | 0.000212887 |
| ENSG00000164627 | KIF6 | -4.850264679 | 1.97E-05 | 0.000213895 |
| ENSG00000198223 | CSF2RA | 3.040751288 | 1.98E-05 | 0.000214686 |
| ENSG00000154678 | PDE1C | -3.616560728 | 2.00E-05 | 0.000216166 |
| ENSG00000211941 | IGHV3-11 | 4.114319601 | 2.02E-05 | 0.000217794 |
| ENSG00000161929 | SCIMP | 3.419880874 | 2.09E-05 | 0.000224938 |
| ENSG00000270885 | RASL10B | -3.672227083 | 2.09E-05 | 0.000224938 |
| ENSG00000196209 | SIRPB2 | 3.460571399 | 2.21E-05 | 0.000236611 |
| ENSG00000169429 | CXCL8 | 3.544488287 | 2.26E-05 | 0.000241526 |
| ENSG00000150625 | GPM6A | -3.875757948 | 2.28E-05 | 0.000243488 |
| ENSG00000112149 | CD83 | 3.334453329 | 2.29E-05 | 0.000243488 |
| ENSG00000279161 | AC093503.3 | -3.272267405 | 2.29E-05 | 0.000243488 |
| ENSG00000103569 | AQP9 | 6.803802794 | 2.35E-05 | 0.00024957 |
| ENSG00000163638 | ADAMTS9 | -3.077184699 | 2.39E-05 | 0.000253168 |
| ENSG00000111262 | KCNA1 | -7.029580462 | 2.39E-05 | 0.000253391 |
| ENSG00000216490 | IFI30 | 3.38575458 | 2.42E-05 | 0.000255138 |
| ENSG00000180767 | CHST13 | 3.831228649 | 2.44E-05 | 0.0002566 |
| ENSG00000128285 | MCHR1 | 4.126810883 | 2.48E-05 | 0.000260258 |
| ENSG00000188176 | SMTNL2 | -4.381413847 | 2.58E-05 | 0.00026941 |
| ENSG00000101188 | NTSR1 | 3.076584443 | 2.64E-05 | 0.000274151 |
| ENSG00000204950 | LRRC10B | -4.281815236 | 2.65E-05 | 0.000275494 |
| ENSG00000174837 | ADGRE1 | 3.336924029 | 2.68E-05 | 0.000277898 |
| ENSG00000261210 | CLEC19A | 4.662965904 | 2.75E-05 | 0.000284636 |
| ENSG00000122121 | XPNPEP2 | -3.300473169 | 2.81E-05 | 0.000289901 |
| ENSG00000229666 | MAST4-AS1 | -3.872074988 | 2.97E-05 | 0.000305169 |
| ENSG00000108576 | SLC6A4 | -5.849617417 | 3.02E-05 | 0.000309659 |
| ENSG00000274414 | AL121772.1 | -3.311767498 | 3.07E-05 | 0.000314149 |
| ENSG00000162670 | BRINP3 | -6.992462554 | 3.09E-05 | 0.000315071 |
| ENSG00000166292 | TMEM100 | -3.805406977 | 3.09E-05 | 0.000315405 |
| ENSG00000264707 | L3MBTL4-AS1 | 3.830178748 | 3.12E-05 | 0.000318207 |
| ENSG00000143001 | TMEM61 | -4.214225851 | 3.14E-05 | 0.000320068 |
| ENSG00000250771 | AC106865.1 | 3.297412996 | 3.14E-05 | 0.000320149 |
| ENSG00000162761 | LMX1A | -6.894584152 | 3.16E-05 | 0.000321021 |
| ENSG00000236939 | BAALC-AS2 | 4.960695212 | 3.19E-05 | 0.000324002 |
| ENSG00000142149 | HUNK | -6.482400545 | 3.19E-05 | 0.000324002 |
| ENSG00000166473 | PKD1L2 | -3.372544462 | 3.19E-05 | 0.00032435 |
| ENSG00000160862 | AZGP1 | -8.023062677 | 3.22E-05 | 0.00032643 |
| ENSG00000268798 | AC027307.3 | -3.210941086 | 3.23E-05 | 0.000327111 |
| ENSG00000169607 | CKAP2L | 3.375904824 | 3.25E-05 | 0.000328467 |
| ENSG00000130528 | HRC | -3.230346417 | 3.36E-05 | 0.000338767 |
| ENSG00000158220 | ESYT3 | -3.312329553 | 3.41E-05 | 0.000343699 |
| ENSG00000226644 | AL121899.1 | 4.54664332 | 3.51E-05 | 0.000352617 |
| ENSG00000206579 | XKR4 | -6.9722831 | 3.61E-05 | 0.000361025 |
| ENSG00000233593 | AL590094.1 | -3.086869474 | 3.72E-05 | 0.000370827 |
| ENSG00000158445 | KCNB1 | -3.680944635 | 3.77E-05 | 0.000375348 |
| ENSG00000141744 | PNMT | -5.103862659 | 3.78E-05 | 0.000376383 |
| ENSG00000254109 | RBPMS-AS1 | -3.096540377 | 3.79E-05 | 0.00037658 |
| ENSG00000148773 | MKI67 | 3.861508598 | 3.97E-05 | 0.000392566 |
| ENSG00000174939 | ASPHD1 | 3.384935319 | 3.98E-05 | 0.000393102 |
| ENSG00000165323 | FAT3 | -3.617734684 | 4.13E-05 | 0.000405917 |
| ENSG00000272908 | AC006033.2 | 3.266382498 | 4.14E-05 | 0.000406545 |
| ENSG00000115353 | TACR1 | -3.080825052 | 4.15E-05 | 0.000406804 |
| ENSG00000260135 | MMP2-AS1 | 4.308605919 | 4.25E-05 | 0.000415396 |
| ENSG00000184601 | C14orf180 | -3.270514004 | 4.29E-05 | 0.000418646 |
| ENSG00000236700 | LINC01010 | 4.671108907 | 4.34E-05 | 0.000423458 |
| ENSG00000226286 | AL008733.1 | -3.557458706 | 4.40E-05 | 0.000428704 |
| ENSG00000205362 | MT1A | -3.277926541 | 4.44E-05 | 0.000431399 |
| ENSG00000113763 | UNC5A | 3.595256329 | 4.45E-05 | 0.000432641 |
| ENSG00000147255 | IGSF1 | -6.448426582 | 4.51E-05 | 0.000437681 |
| ENSG00000211666 | IGLV2-14 | 3.105354067 | 4.59E-05 | 0.000444676 |
| ENSG00000119457 | SLC46A2 | 3.695919415 | 4.65E-05 | 0.000449418 |
| ENSG00000250548 | LINC01303 | 3.209662853 | 4.65E-05 | 0.000449419 |
| ENSG00000153253 | SCN3A | -4.523413399 | 4.69E-05 | 0.000452368 |
| ENSG00000125851 | PCSK2 | -6.688055115 | 4.74E-05 | 0.000456934 |
| ENSG00000135333 | EPHA7 | -4.199991062 | 5.15E-05 | 0.000492635 |
| ENSG00000196167 | COLCA1 | -3.152622461 | 5.18E-05 | 0.00049423 |
| ENSG00000177990 | DPY19L2 | -3.350706276 | 5.25E-05 | 0.000499845 |
| ENSG00000111432 | FZD10 | -3.646597904 | 5.37E-05 | 0.00051014 |
| ENSG00000125848 | FLRT3 | -3.2867359 | 5.43E-05 | 0.000514533 |
| ENSG00000156970 | BUB1B | 3.223893374 | 5.58E-05 | 0.000527228 |
| ENSG00000170381 | SEMA3E | -6.661912326 | 5.68E-05 | 0.00053643 |
| ENSG00000211933 | IGHV6-1 | 4.248230081 | 5.77E-05 | 0.000543461 |
| ENSG00000158428 | CATIP | 3.513983286 | 5.78E-05 | 0.000544718 |
| ENSG00000106004 | HOXA5 | -4.527919939 | 5.79E-05 | 0.000544868 |
| ENSG00000250378 | AC114296.1 | 5.835946535 | 5.80E-05 | 0.000545352 |
| ENSG00000225028 | AC096541.1 | -4.623710907 | 6.24E-05 | 0.00058289 |
| ENSG00000198353 | HOXC4 | -3.733506678 | 6.34E-05 | 0.000589838 |
| ENSG00000226031 | FGF13-AS1 | -3.583682852 | 6.61E-05 | 0.000613392 |
| ENSG00000130032 | PRRG3 | -4.760641488 | 6.63E-05 | 0.000614754 |
| ENSG00000142748 | FCN3 | -3.145078549 | 6.63E-05 | 0.000614754 |
| ENSG00000254489 | MPPED2-AS1 | -4.972033574 | 6.64E-05 | 0.000614875 |
| ENSG00000120729 | MYOT | -3.318025884 | 6.70E-05 | 0.000620087 |
| ENSG00000268581 | SIGLEC18P | 4.654510364 | 6.80E-05 | 0.000627026 |
| ENSG00000101198 | NKAIN4 | 3.669040465 | 7.23E-05 | 0.000662615 |
| ENSG00000239855 | IGKV1-6 | 4.030908658 | 7.71E-05 | 0.000701228 |
| ENSG00000225472 | AL136366.1 | -3.158201576 | 7.99E-05 | 0.000723869 |
| ENSG00000137558 | PI15 | -3.675521932 | 8.08E-05 | 0.000730728 |
| ENSG00000060718 | COL11A1 | 6.813739286 | 8.09E-05 | 0.000730998 |
| ENSG00000225706 | PTPRD-AS1 | -3.498900137 | 8.66E-05 | 0.00077701 |
| ENSG00000196468 | FGF16 | -6.314861971 | 8.98E-05 | 0.000801227 |
| ENSG00000130700 | GATA5 | -3.481479641 | 9.01E-05 | 0.000803533 |
| ENSG00000158164 | TMSB15A | -4.749227897 | 9.03E-05 | 0.000804118 |
| ENSG00000196664 | TLR7 | 3.355705628 | 9.11E-05 | 0.000809695 |
| ENSG00000259094 | AC013457.1 | 6.829430545 | 9.25E-05 | 0.000820256 |
| ENSG00000117399 | CDC20 | 3.013904841 | 9.33E-05 | 0.000827196 |
| ENSG00000137699 | TRIM29 | -4.339447914 | 9.43E-05 | 0.000834807 |
| ENSG00000261105 | LMO7-AS1 | -3.321728472 | 9.52E-05 | 0.00084247 |
| ENSG00000073737 | DHRS9 | 3.211634566 | 9.55E-05 | 0.000844256 |
| ENSG00000285018 | AC245140.3 | -3.815861015 | 9.66E-05 | 0.000852182 |
| ENSG00000204044 | SLC12A5-AS1 | 6.298570244 | 9.87E-05 | 0.000867632 |
| ENSG00000119698 | PPP4R4 | -4.366027549 | 9.91E-05 | 0.000871211 |
| ENSG00000272899 | ATP6V1FNB | 3.440210866 | 9.94E-05 | 0.000873864 |
| ENSG00000284391 | AL139398.1 | -3.111044106 | 9.96E-05 | 0.000874462 |
| ENSG00000160223 | ICOSLG | 3.079127982 | 0.00010077 | 0.000883438 |
| ENSG00000226816 | AC005082.1 | 3.102483492 | 0.000106563 | 0.000929874 |
| ENSG00000169435 | RASSF6 | 4.169559725 | 0.000106959 | 0.000932604 |
| ENSG00000196917 | HCAR1 | -4.822318302 | 0.000107975 | 0.000940367 |
| ENSG00000225670 | CADM3-AS1 | -3.347083639 | 0.000110604 | 0.000961776 |
| ENSG00000230526 | AC073370.1 | 6.006062335 | 0.000114347 | 0.000991639 |
| ENSG00000274276 | CBSL | -3.462611546 | 0.000114662 | 0.000993986 |
| ENSG00000091482 | SMPX | -6.456431699 | 0.000117575 | 0.0010165 |
| ENSG00000170627 | GTSF1 | 3.000852644 | 0.000118091 | 0.001020175 |
| ENSG00000280323 | AC053503.6 | -6.586287137 | 0.000119935 | 0.001032927 |
| ENSG00000230712 | GGTLC4P | -3.905786786 | 0.000121254 | 0.001043093 |
| ENSG00000091704 | CPA1 | -6.439286151 | 0.000127879 | 0.001093808 |
| ENSG00000088882 | CPXM1 | -3.504728037 | 0.000133306 | 0.001135043 |
| ENSG00000166926 | MS4A6E | 6.434436485 | 0.000135603 | 0.001150353 |
| ENSG00000126787 | DLGAP5 | 3.600023435 | 0.000136832 | 0.001158647 |
| ENSG00000248874 | C5orf17 | 3.970653254 | 0.000137817 | 0.001165513 |
| ENSG00000103184 | SEC14L5 | -5.996075424 | 0.000138244 | 0.001167802 |
| ENSG00000233817 | AL162727.1 | -6.596085157 | 0.000139477 | 0.001177336 |
| ENSG00000197757 | HOXC6 | -5.837033363 | 0.000139874 | 0.001179569 |
| ENSG00000171903 | CYP4F11 | -3.199390764 | 0.000140432 | 0.001183179 |
| ENSG00000109193 | SULT1E1 | 5.912588712 | 0.000141422 | 0.001191068 |
| ENSG00000185290 | NUPR2 | -4.334381844 | 0.000142624 | 0.001199843 |
| ENSG00000009790 | TRAF3IP3 | 3.035386029 | 0.000143177 | 0.001202697 |
| ENSG00000211676 | IGLJ2 | 6.579850995 | 0.000147183 | 0.001231746 |
| ENSG00000226197 | AL583785.1 | -4.083818717 | 0.000158612 | 0.001314187 |
| ENSG00000181092 | ADIPOQ | -10.29526155 | 0.000163577 | 0.001352837 |
| ENSG00000197406 | DIO3 | -5.043476926 | 0.000165615 | 0.001367174 |
| ENSG00000204971 | AP000812.1 | 5.019760368 | 0.000166468 | 0.001371405 |
| ENSG00000211716 | TRBV9 | 4.361007668 | 0.000167048 | 0.001375467 |
| ENSG00000162631 | NTNG1 | -4.138465621 | 0.000168622 | 0.001385893 |
| ENSG00000248187 | AC078850.1 | 3.813578106 | 0.00017195 | 0.001410153 |
| ENSG00000279302 | AC013643.3 | -6.407343254 | 0.000174089 | 0.001426132 |
| ENSG00000176083 | ZNF683 | 3.423393006 | 0.000183358 | 0.001496616 |
| ENSG00000119946 | CNNM1 | -3.125438286 | 0.000183592 | 0.001497978 |
| ENSG00000127252 | HRASLS | -5.85624675 | 0.000184261 | 0.001502895 |
| ENSG00000223774 | AL513217.1 | -3.665642694 | 0.000188523 | 0.001532968 |
| ENSG00000198074 | AKR1B10 | -5.75892784 | 0.000189186 | 0.001536934 |
| ENSG00000183742 | MACC1 | 3.402304238 | 0.000192117 | 0.001557932 |
| ENSG00000183807 | FAM162B | -3.464011883 | 0.000195917 | 0.001584018 |
| ENSG00000162892 | IL24 | 3.181345662 | 0.000197373 | 0.001593652 |
| ENSG00000245598 | DACT3-AS1 | -3.563475754 | 0.000197872 | 0.001597108 |
| ENSG00000225107 | AC092484.1 | 6.683397052 | 0.000198032 | 0.001597831 |
| ENSG00000237742 | AL365259.1 | -3.759514838 | 0.000199476 | 0.001606018 |
| ENSG00000260880 | HCCAT5 | -4.644242125 | 0.00020925 | 0.00167452 |
| ENSG00000186818 | LILRB4 | 4.55731632 | 0.000210029 | 0.001679558 |
| ENSG00000214456 | PLIN5 | -4.038087071 | 0.000211483 | 0.001687586 |
| ENSG00000146013 | GFRA3 | -4.868698681 | 0.000214489 | 0.001709821 |
| ENSG00000161649 | CD300LG | -4.278985142 | 0.000214803 | 0.001711043 |
| ENSG00000143171 | RXRG | -5.826830012 | 0.000215524 | 0.001714966 |
| ENSG00000211962 | IGHV1-46 | 3.473809386 | 0.00021729 | 0.001727185 |
| ENSG00000280165 | PCDH20 | -6.415554484 | 0.000221681 | 0.001759601 |
| ENSG00000232810 | TNF | 3.23622127 | 0.000222171 | 0.001762245 |
| ENSG00000178773 | CPNE7 | 3.219308108 | 0.000224207 | 0.001774636 |
| ENSG00000234869 | AL021392.1 | 3.642942953 | 0.000228864 | 0.001807678 |
| ENSG00000187151 | ANGPTL5 | -3.251420279 | 0.000233142 | 0.001836949 |
| ENSG00000080293 | SCTR | 3.289905066 | 0.000236039 | 0.001856529 |
| ENSG00000260757 | AC093520.1 | 5.73474615 | 0.000240112 | 0.001883201 |
| ENSG00000225937 | PCA3 | -5.00480201 | 0.000247939 | 0.001936328 |
| ENSG00000265975 | AC002091.1 | 3.796764216 | 0.000256418 | 0.001997945 |
| ENSG00000198099 | ADH4 | -3.847715848 | 0.000259789 | 0.002022618 |
| ENSG00000165269 | AQP7 | -4.313996647 | 0.000275658 | 0.002131772 |
| ENSG00000130226 | DPP6 | 4.485608983 | 0.000275918 | 0.002132883 |
| ENSG00000259717 | LINC00677 | 4.246074573 | 0.00027872 | 0.002152326 |
| ENSG00000054179 | ENTPD2 | -6.214047636 | 0.000279813 | 0.002160025 |
| ENSG00000259134 | LINC00924 | -3.098618687 | 0.000294802 | 0.002262527 |
| ENSG00000251364 | AC107884.1 | 3.048903751 | 0.000295001 | 0.002263284 |
| ENSG00000249679 | AC106897.1 | -3.395677168 | 0.000302966 | 0.002315704 |
| ENSG00000276775 | IGHV4-4 | 4.457733211 | 0.000304805 | 0.002328176 |
| ENSG00000253369 | AC131902.1 | -6.396687173 | 0.000305703 | 0.002334243 |
| ENSG00000166819 | PLIN1 | -5.739773605 | 0.00031347 | 0.002390305 |
| ENSG00000165548 | TMEM63C | 4.610404288 | 0.000313603 | 0.002390504 |
| ENSG00000272839 | AC011899.3 | 4.968421021 | 0.000313909 | 0.002392029 |
| ENSG00000244116 | IGKV2-28 | 3.3707647 | 0.000317603 | 0.00241772 |
| ENSG00000124253 | PCK1 | -8.896915851 | 0.000318206 | 0.002421491 |
| ENSG00000277851 | LINC02391 | 3.898205035 | 0.000321046 | 0.002438972 |
| ENSG00000163873 | GRIK3 | -5.887745282 | 0.000324576 | 0.002464127 |
| ENSG00000204175 | GPRIN2 | -7.058362394 | 0.000333977 | 0.002525267 |
| ENSG00000248477 | AC139495.1 | 3.479732174 | 0.000334766 | 0.002529532 |
| ENSG00000089225 | TBX5 | 4.66023214 | 0.000338981 | 0.002558801 |
| ENSG00000233028 | AC006970.2 | -5.892083018 | 0.000344263 | 0.002592582 |
| ENSG00000166866 | MYO1A | 3.368802461 | 0.000345301 | 0.002599522 |
| ENSG00000279384 | AC080188.2 | -3.206558352 | 0.00035589 | 0.0026703 |
| ENSG00000255741 | AP000808.2 | -4.712387597 | 0.000357638 | 0.002680732 |
| ENSG00000211753 | TRBV28 | 3.404400901 | 0.000366779 | 0.002736414 |
| ENSG00000165379 | LRFN5 | -3.897702572 | 0.000372218 | 0.002766966 |
| ENSG00000251504 | LINC01099 | -6.410260931 | 0.000375534 | 0.002785172 |
| ENSG00000074803 | SLC12A1 | -4.44122636 | 0.000376544 | 0.002791737 |
| ENSG00000016402 | IL20RA | -6.640138639 | 0.000376974 | 0.002794007 |
| ENSG00000160808 | MYL3 | -3.385613937 | 0.000379753 | 0.002812751 |
| ENSG00000145808 | ADAMTS19 | -5.631885971 | 0.000383306 | 0.00283533 |
| ENSG00000102174 | PHEX | -3.02718646 | 0.000383837 | 0.002838326 |
| ENSG00000167941 | SOST | -5.362614305 | 0.000392331 | 0.002898276 |
| ENSG00000236404 | VLDLR-AS1 | -3.069000572 | 0.000393895 | 0.002908247 |
| ENSG00000243810 | AL121721.1 | 3.933146656 | 0.000413936 | 0.003040896 |
| ENSG00000130208 | APOC1 | 5.614973658 | 0.000414317 | 0.0030427 |
| ENSG00000237949 | LINC00844 | -3.522173587 | 0.000419273 | 0.00307508 |
| ENSG00000249639 | AC022092.1 | 6.065391211 | 0.000423642 | 0.003100048 |
| ENSG00000234147 | AL035446.1 | 3.335851098 | 0.000430076 | 0.003137939 |
| ENSG00000268849 | SIGLEC22P | 3.020110805 | 0.000433742 | 0.003161605 |
| ENSG00000167434 | CA4 | -4.751620761 | 0.000441378 | 0.003211024 |
| ENSG00000173991 | TCAP | -3.083512099 | 0.000443255 | 0.00322051 |
| ENSG00000232892 | AL499616.1 | -6.163926018 | 0.000445303 | 0.0032333 |
| ENSG00000110680 | CALCA | -5.446137594 | 0.000446014 | 0.003236372 |
| ENSG00000159261 | CLDN14 | 3.472351637 | 0.000451128 | 0.003268208 |
| ENSG00000196611 | MMP1 | 8.672161132 | 0.000453223 | 0.00328127 |
| ENSG00000224420 | ADM5 | -3.339006534 | 0.000454374 | 0.003285769 |
| ENSG00000232046 | LINC01798 | -4.345688142 | 0.000454429 | 0.003285769 |
| ENSG00000228742 | LINC02577 | 6.305065823 | 0.000473685 | 0.003408563 |
| ENSG00000164935 | DCSTAMP | 6.042287374 | 0.000474375 | 0.003412436 |
| ENSG00000175879 | HOXD8 | -3.234563157 | 0.000474729 | 0.003412839 |
| ENSG00000128965 | CHAC1 | -3.102675308 | 0.000474735 | 0.003412839 |
| ENSG00000225026 | AC091492.1 | 5.203620135 | 0.000478068 | 0.003432412 |
| ENSG00000266274 | RN7SL138P | 3.033976119 | 0.000478689 | 0.003434797 |
| ENSG00000237517 | DGCR5 | 4.386329699 | 0.000478835 | 0.003434797 |
| ENSG00000078900 | TP73 | 3.285156049 | 0.000479963 | 0.003441334 |
| ENSG00000133063 | CHIT1 | 7.649642367 | 0.000487422 | 0.00348738 |
| ENSG00000136944 | LMX1B | -3.299460486 | 0.000500005 | 0.003567138 |
| ENSG00000204099 | NEU4 | 3.392383585 | 0.000505094 | 0.003597736 |
| ENSG00000166415 | WDR72 | -7.026447098 | 0.000519599 | 0.003694034 |
| ENSG00000145506 | NKD2 | -4.692102369 | 0.000531353 | 0.003768065 |
| ENSG00000250310 | AC006487.2 | -6.186616871 | 0.000538351 | 0.003812881 |
| ENSG00000102575 | ACP5 | 5.008843718 | 0.000549389 | 0.003884937 |
| ENSG00000081051 | AFP | -6.13053229 | 0.000553863 | 0.003911654 |
| ENSG00000144290 | SLC4A10 | 3.400106808 | 0.000570441 | 0.004016123 |
| ENSG00000111245 | MYL2 | -5.463563703 | 0.000575915 | 0.004048329 |
| ENSG00000171819 | ANGPTL7 | -3.44397572 | 0.000584291 | 0.00409992 |
| ENSG00000178752 | ERFE | -3.14126425 | 0.000592401 | 0.004144781 |
| ENSG00000147509 | RGS20 | 3.139980218 | 0.000618306 | 0.004303261 |
| ENSG00000183775 | KCTD16 | -3.000108814 | 0.000628104 | 0.004370103 |
| ENSG00000144331 | ZNF385B | -3.712917182 | 0.000638564 | 0.004433277 |
| ENSG00000233005 | AC018742.1 | -3.001746159 | 0.000654988 | 0.004536101 |
| ENSG00000143452 | HORMAD1 | 5.536655058 | 0.00068215 | 0.004695286 |
| ENSG00000184661 | CDCA2 | 3.135645813 | 0.000683076 | 0.004698782 |
| ENSG00000282840 | AC004554.2 | -6.22199772 | 0.000686989 | 0.004719925 |
| ENSG00000112175 | BMP5 | -5.950767148 | 0.000690822 | 0.004741912 |
| ENSG00000056487 | PHF21B | -6.002409206 | 0.000694076 | 0.004758439 |
| ENSG00000269553 | U62631.1 | 5.027403598 | 0.000715495 | 0.004888889 |
| ENSG00000258867 | LINC01146 | 3.0013025 | 0.000720895 | 0.004916823 |
| ENSG00000109805 | NCAPG | 3.011816568 | 0.000722085 | 0.004923449 |
| ENSG00000258987 | AL132642.1 | -3.18313573 | 0.000722884 | 0.004927401 |
| ENSG00000080224 | EPHA6 | -6.609048699 | 0.000730378 | 0.004969446 |
| ENSG00000105697 | HAMP | 5.675723421 | 0.00073704 | 0.005008712 |
| ENSG00000259225 | LINC02345 | 3.374000587 | 0.00074564 | 0.005059507 |
| ENSG00000211945 | IGHV1-18 | 3.383094725 | 0.000746196 | 0.005060647 |
| ENSG00000244558 | KCNK15-AS1 | -3.194395794 | 0.000746258 | 0.005060647 |
| ENSG00000228013 | IL6R-AS1 | 3.124954755 | 0.000749759 | 0.005078075 |
| ENSG00000228668 | TRGV5P | 4.219663079 | 0.00075719 | 0.005120883 |
| ENSG00000280193 | AC132219.2 | -3.197954517 | 0.000774221 | 0.005220362 |
| ENSG00000146038 | DCDC2 | 3.469669385 | 0.000783459 | 0.005274743 |
| ENSG00000102837 | OLFM4 | -4.537886693 | 0.000787961 | 0.005301877 |
| ENSG00000255400 | AC124276.2 | -3.573085207 | 0.000809889 | 0.00542993 |
| ENSG00000164161 | HHIP | -3.607315461 | 0.000816006 | 0.005466046 |
| ENSG00000145248 | SLC10A4 | -3.358285523 | 0.000821585 | 0.005500141 |
| ENSG00000283992 | SLURP2 | -5.965742085 | 0.000824903 | 0.005519072 |
| ENSG00000211966 | IGHV5-51 | 3.368013714 | 0.000827365 | 0.00553236 |
| ENSG00000196277 | GRM7 | -4.540222488 | 0.000832597 | 0.005558967 |
| ENSG00000239961 | LILRA4 | 6.201052985 | 0.000871527 | 0.005781131 |
| ENSG00000256262 | USP30-AS1 | 3.443641238 | 0.000886152 | 0.005864706 |
| ENSG00000214076 | CPSF1P1 | 3.855374532 | 0.000886412 | 0.005864706 |
| ENSG00000205358 | MT1H | 3.855605572 | 0.000889588 | 0.005878389 |
| ENSG00000177602 | HASPIN | 3.697305588 | 0.000901726 | 0.005945182 |
| ENSG00000185352 | HS6ST3 | -6.672712857 | 0.000902075 | 0.005945182 |
| ENSG00000232258 | TMEM114 | 5.008319374 | 0.000908309 | 0.005982761 |
| ENSG00000259354 | AC025580.2 | 4.355500368 | 0.000920463 | 0.006052176 |
| ENSG00000145107 | TM4SF19 | 6.287869769 | 0.000929969 | 0.006107538 |
| ENSG00000211662 | IGLV3-21 | 5.329983725 | 0.000942494 | 0.006180772 |
| ENSG00000277351 | AC013553.3 | -3.03619591 | 0.000957547 | 0.00626852 |
| ENSG00000042062 | RIPOR3 | -3.000966915 | 0.000960454 | 0.006285717 |
| ENSG00000250697 | AC010343.3 | 4.220659894 | 0.000971538 | 0.006349022 |
| ENSG00000177354 | C10orf71 | -5.955790263 | 0.000974577 | 0.006367027 |
| ENSG00000224557 | HLA-DPB2 | 3.067624135 | 0.001000249 | 0.006500753 |
| ENSG00000072571 | HMMR | 3.167809838 | 0.001001744 | 0.006508584 |
| ENSG00000124140 | SLC12A5 | 3.460929667 | 0.001004677 | 0.006523954 |
| ENSG00000130164 | LDLR | -4.181778841 | 0.001016005 | 0.006585596 |
| ENSG00000240864 | IGKV1-16 | 3.51802297 | 0.001021971 | 0.006617063 |
| ENSG00000269446 | AC006967.3 | 6.141443062 | 0.001031861 | 0.00667341 |
| ENSG00000118160 | SLC8A2 | -5.218447409 | 0.00103844 | 0.006710174 |
| ENSG00000140297 | GCNT3 | 3.62332692 | 0.001045677 | 0.006747241 |
| ENSG00000170373 | CST1 | 5.483562196 | 0.001056327 | 0.006802295 |
| ENSG00000115008 | IL1A | 3.899641366 | 0.001075136 | 0.006899709 |
| ENSG00000004948 | CALCR | 3.763064069 | 0.001077956 | 0.006915831 |
| ENSG00000007314 | SCN4A | -3.730794569 | 0.001082139 | 0.006940692 |
| ENSG00000274461 | AL391425.1 | 3.559651845 | 0.001113757 | 0.007111034 |
| ENSG00000164047 | CAMP | 3.586516981 | 0.001123749 | 0.007164659 |
| ENSG00000183535 | COL18A1-AS1 | -3.295097004 | 0.001136826 | 0.007239822 |
| ENSG00000182050 | MGAT4C | -3.838502382 | 0.001138119 | 0.007242226 |
| ENSG00000183798 | EMILIN3 | -5.648534946 | 0.001141887 | 0.007263822 |
| ENSG00000214846 | AC114744.1 | 3.474069144 | 0.001151037 | 0.007313754 |
| ENSG00000005001 | PRSS22 | 5.004945142 | 0.001151785 | 0.00731644 |
| ENSG00000135218 | CD36 | 4.059456678 | 0.001153686 | 0.007324373 |
| ENSG00000144481 | TRPM8 | 4.314359143 | 0.001161459 | 0.007365409 |
| ENSG00000179399 | GPC5 | -3.559397792 | 0.001207307 | 0.007616056 |
| ENSG00000122254 | HS3ST2 | 4.567882623 | 0.001215585 | 0.007652533 |
| ENSG00000187140 | FOXD3 | -5.971497198 | 0.001231393 | 0.007738752 |
| ENSG00000163993 | S100P | -3.643735449 | 0.001236736 | 0.007768293 |
| ENSG00000179344 | HLA-DQB1 | 5.800627954 | 0.001240219 | 0.007785817 |
| ENSG00000230699 | AL645608.2 | -6.53247648 | 0.00124761 | 0.007823483 |
| ENSG00000220378 | KRT8P42 | 6.312979027 | 0.001293725 | 0.008069898 |
| ENSG00000211965 | IGHV3-49 | 3.554058411 | 0.001305022 | 0.008122345 |
| ENSG00000251546 | IGKV1D-39 | 3.413818482 | 0.001316503 | 0.008184739 |
| ENSG00000039068 | CDH1 | 3.864799198 | 0.00135116 | 0.008365513 |
| ENSG00000250687 | AC146944.2 | 4.832287555 | 0.001362191 | 0.008429165 |
| ENSG00000105610 | KLF1 | 6.044788152 | 0.001371002 | 0.008467378 |
| ENSG00000188338 | SLC38A3 | -5.786094482 | 0.00137282 | 0.008474612 |
| ENSG00000082556 | OPRK1 | -5.993059183 | 0.001394797 | 0.008578991 |
| ENSG00000274818 | AC004825.2 | -3.234474955 | 0.001414084 | 0.008666076 |
| ENSG00000187123 | LYPD6 | -5.240281661 | 0.001416832 | 0.008676551 |
| ENSG00000064787 | BCAS1 | -5.485387372 | 0.001426695 | 0.008729819 |
| ENSG00000143632 | ACTA1 | -3.203362915 | 0.001438546 | 0.008797545 |
| ENSG00000263812 | LINC00908 | -5.758499369 | 0.001453285 | 0.008873205 |
| ENSG00000251379 | AC099550.1 | -5.758499369 | 0.001453285 | 0.008873205 |
| ENSG00000211950 | IGHV1-24 | 4.227006689 | 0.0014578 | 0.008898356 |
| ENSG00000164142 | FAM160A1 | -3.805114339 | 0.001460214 | 0.008905839 |
| ENSG00000137675 | MMP27 | -5.973937273 | 0.001474207 | 0.008983874 |
| ENSG00000198796 | ALPK2 | 5.921661739 | 0.001540242 | 0.009340726 |
| ENSG00000196169 | KIF19 | -3.001142119 | 0.001571564 | 0.00951016 |
| ENSG00000158022 | TRIM63 | 4.233548723 | 0.001595858 | 0.009639015 |
| ENSG00000167414 | GNG8 | -3.301704897 | 0.001609611 | 0.009711653 |
| ENSG00000146151 | HMGCLL1 | -5.819919847 | 0.001622769 | 0.009772685 |
| ENSG00000165131 | LLCFC1 | 5.476935431 | 0.001629983 | 0.009810872 |
| ENSG00000260838 | AC022893.2 | -4.366700779 | 0.00167139 | 0.010027895 |
| ENSG00000167476 | JSRP1 | 3.759808413 | 0.001678163 | 0.010055117 |
| ENSG00000141665 | FBXO15 | 3.332554912 | 0.001708006 | 0.010214875 |
| ENSG00000131771 | PPP1R1B | -4.824156317 | 0.001713938 | 0.010244905 |
| ENSG00000154319 | FAM167A | 3.646477586 | 0.00175693 | 0.01046849 |
| ENSG00000132185 | FCRLA | 3.461303918 | 0.001758264 | 0.010473661 |
| ENSG00000181449 | SOX2 | -6.056329765 | 0.001779348 | 0.010576841 |
| ENSG00000265415 | AC099850.3 | 3.363431047 | 0.001812649 | 0.010752052 |
| ENSG00000225101 | OR52K3P | 3.497468776 | 0.001839472 | 0.010891048 |
| ENSG00000135253 | KCP | 4.417365557 | 0.001842277 | 0.010904787 |
| ENSG00000146070 | PLA2G7 | 4.783382309 | 0.001852713 | 0.010955021 |
| ENSG00000156920 | ADGRG4 | -5.93558939 | 0.001886015 | 0.011125606 |
| ENSG00000171848 | RRM2 | 3.419574376 | 0.001891438 | 0.011151747 |
| ENSG00000179593 | ALOX15B | 4.856034941 | 0.001922854 | 0.011283722 |
| ENSG00000272944 | AC079834.2 | -5.239456479 | 0.001934693 | 0.011338398 |
| ENSG00000259430 | CERS3-AS1 | -5.115849894 | 0.001941351 | 0.011362967 |
| ENSG00000275385 | CCL18 | 5.744354865 | 0.001941411 | 0.011362967 |
| ENSG00000144583 | 4-Mar | 3.224072389 | 0.001949039 | 0.011401678 |
| ENSG00000152931 | PART1 | -4.508253261 | 0.001956449 | 0.011439422 |
| ENSG00000273001 | AL731533.2 | -4.127517428 | 0.001968222 | 0.011475087 |
| ENSG00000186583 | SPATC1 | 3.218279889 | 0.001977518 | 0.011507224 |
| ENSG00000260186 | LINC02137 | 5.94141423 | 0.001983665 | 0.011529254 |
| ENSG00000101850 | GPR143 | 3.479797576 | 0.001993211 | 0.011572768 |
| ENSG00000261706 | LINC00165 | -3.812746056 | 0.001998601 | 0.011595084 |
| ENSG00000270638 | AL023806.1 | -5.717211944 | 0.002016032 | 0.011666113 |
| ENSG00000236841 | AC007750.1 | 3.030669415 | 0.002050804 | 0.011833151 |
| ENSG00000182077 | PTCHD3 | 5.586240738 | 0.002054379 | 0.011848377 |
| ENSG00000181234 | TMEM132C | -4.211133161 | 0.00205505 | 0.011849207 |
| ENSG00000171246 | NPTX1 | -4.036455242 | 0.002090486 | 0.012019627 |
| ENSG00000228216 | AL355607.1 | -5.342571487 | 0.002103957 | 0.012093987 |
| ENSG00000133055 | MYBPH | 3.752712302 | 0.002139435 | 0.012275959 |
| ENSG00000237152 | DLEU7-AS1 | 3.9040191 | 0.002145504 | 0.012301657 |
| ENSG00000225216 | AC007362.1 | 3.100634794 | 0.002149612 | 0.012316336 |
| ENSG00000147676 | MAL2 | -5.463581212 | 0.002184339 | 0.012473139 |
| ENSG00000278030 | TRBV7-9 | 3.783980603 | 0.002208872 | 0.012597235 |
| ENSG00000121207 | LRAT | -5.774216029 | 0.002211581 | 0.01260949 |
| ENSG00000120659 | TNFSF11 | 4.527792423 | 0.002215878 | 0.012621188 |
| ENSG00000274977 | AC011298.2 | 5.354213742 | 0.002239313 | 0.012738534 |
| ENSG00000185985 | SLITRK2 | -3.619500593 | 0.002249057 | 0.012781032 |
| ENSG00000203877 | RIPPLY2 | -5.891411429 | 0.00230886 | 0.013071335 |
| ENSG00000259744 | AC009269.4 | -5.782446609 | 0.002338415 | 0.013228661 |
| ENSG00000206384 | COL6A6 | -3.42409734 | 0.002352168 | 0.01329643 |
| ENSG00000203837 | PNLIPRP3 | -5.915251982 | 0.002362408 | 0.013337553 |
| ENSG00000269933 | AL031429.2 | -6.033750859 | 0.002363223 | 0.013338807 |
| ENSG00000230852 | AL161621.1 | -5.033630398 | 0.002384745 | 0.013431005 |
| ENSG00000006047 | YBX2 | -4.923172779 | 0.002415169 | 0.013584271 |
| ENSG00000227471 | AKR1B15 | -3.563646496 | 0.002470757 | 0.013844981 |
| ENSG00000145832 | SLC25A48 | 3.692824545 | 0.002504957 | 0.014008697 |
| ENSG00000092067 | CEBPE | 3.342456607 | 0.002506726 | 0.014013947 |
| ENSG00000111783 | RFX4 | 4.075040234 | 0.002541205 | 0.014183193 |
| ENSG00000237111 | IGHJ3P | 4.099673099 | 0.002563398 | 0.014291416 |
| ENSG00000234840 | LINC01239 | -6.017008224 | 0.002591591 | 0.014425026 |
| ENSG00000176194 | CIDEA | -5.566838545 | 0.002605372 | 0.014491589 |
| ENSG00000213088 | ACKR1 | -3.914961113 | 0.002605483 | 0.014491589 |
| ENSG00000174607 | UGT8 | -3.629163579 | 0.002609889 | 0.014508919 |
| ENSG00000148735 | PLEKHS1 | 3.351775962 | 0.002619911 | 0.014561031 |
| ENSG00000135094 | SDS | 4.530154699 | 0.002621866 | 0.014564699 |
| ENSG00000234919 | LINC01827 | 4.227637339 | 0.002650416 | 0.014697882 |
| ENSG00000230393 | AC092667.1 | -5.828866298 | 0.002661342 | 0.014740293 |
| ENSG00000230061 | TRPM2-AS | 3.379176162 | 0.002664612 | 0.014748958 |
| ENSG00000095203 | EPB41L4B | -3.841573993 | 0.002677299 | 0.014803155 |
| ENSG00000228791 | THRB-AS1 | -3.6832012 | 0.00274016 | 0.015106149 |
| ENSG00000248869 | LINC02511 | -5.685149231 | 0.002779751 | 0.015290669 |
| ENSG00000211956 | IGHV4-34 | 3.435554577 | 0.002797534 | 0.015354684 |
| ENSG00000196242 | OR2C3 | 5.204653052 | 0.002808052 | 0.015393627 |
| ENSG00000254533 | AF186192.1 | -5.617645381 | 0.002834688 | 0.015505627 |
| ENSG00000232363 | AL021391.1 | -5.488972122 | 0.002913233 | 0.015858113 |
| ENSG00000211976 | IGHV3-73 | 3.907828492 | 0.00291964 | 0.0158853 |
| ENSG00000276566 | IGKV1D-13 | 5.23319376 | 0.00293875 | 0.015966093 |
| ENSG00000250007 | AC087457.1 | -5.643246702 | 0.002943907 | 0.015978674 |
| ENSG00000271216 | LINC01050 | 8.64373203 | 0.002966084 | 0.016079272 |
| ENSG00000114279 | FGF12 | -3.909726233 | 0.002967909 | 0.016079272 |
| ENSG00000239467 | AC007405.3 | -3.984569736 | 0.002995891 | 0.016213862 |
| ENSG00000172058 | SERF1A | -3.822237085 | 0.002997439 | 0.016217156 |
| ENSG00000211937 | IGHV2-5 | 3.124821358 | 0.003051239 | 0.016454005 |
| ENSG00000285731 | AC090644.1 | -3.631379513 | 0.003060164 | 0.016494224 |
| ENSG00000257336 | PRELID2P1 | 5.19927028 | 0.003068915 | 0.016525549 |
| ENSG00000198944 | SOWAHA | -3.095314859 | 0.003087839 | 0.016608517 |
| ENSG00000250327 | RPSAP70 | -3.550629569 | 0.003088754 | 0.016608517 |
| ENSG00000285569 | AC099344.3 | -4.018470301 | 0.003148162 | 0.016887577 |
| ENSG00000183783 | KCTD8 | -3.530870232 | 0.003160283 | 0.016948552 |
| ENSG00000242048 | AC093583.1 | 4.759998633 | 0.003273181 | 0.017462399 |
| ENSG00000268758 | ADGRE4P | 3.147162288 | 0.003294302 | 0.017541788 |
| ENSG00000105366 | SIGLEC8 | 6.107812181 | 0.003295296 | 0.017542926 |
| ENSG00000240654 | C1QTNF9 | -4.952409493 | 0.003327378 | 0.017688591 |
| ENSG00000202000 | RNU1-36P | -5.620357192 | 0.003330604 | 0.017699979 |
| ENSG00000173597 | SULT1B1 | 3.21337333 | 0.003331094 | 0.017699979 |
| ENSG00000152779 | SLC16A12 | -3.025691084 | 0.003408432 | 0.018053961 |
| ENSG00000125900 | SIRPD | 4.618159503 | 0.003429231 | 0.018144263 |
| ENSG00000268297 | CLEC4GP1 | -4.152329453 | 0.003448637 | 0.018221219 |
| ENSG00000211794 | TRAV12-3 | 4.670471778 | 0.003460936 | 0.01827761 |
| ENSG00000173406 | DAB1 | -4.9750425 | 0.003479113 | 0.01836067 |
| ENSG00000184408 | KCND2 | -3.92535608 | 0.003480147 | 0.018361818 |
| ENSG00000136531 | SCN2A | -3.230014687 | 0.003540001 | 0.018612112 |
| ENSG00000101746 | NOL4 | -5.552076084 | 0.003607939 | 0.018903013 |
| ENSG00000147614 | ATP6V0D2 | 6.55351989 | 0.003625483 | 0.018989104 |
| ENSG00000284719 | AL033527.5 | 3.77102853 | 0.003626898 | 0.018989104 |
| ENSG00000261701 | HPR | -4.505541572 | 0.003649079 | 0.019087424 |
| ENSG00000197272 | IL27 | 3.06205589 | 0.00380689 | 0.019774897 |
| ENSG00000227141 | AL160286.1 | -5.103210728 | 0.003814455 | 0.019805047 |
| ENSG00000261026 | AC105046.1 | -3.650237511 | 0.003851637 | 0.01996583 |
| ENSG00000166426 | CRABP1 | -5.868715766 | 0.003854977 | 0.019978537 |
| ENSG00000218537 | MIF-AS1 | -3.080997726 | 0.003871899 | 0.020042127 |
| ENSG00000104435 | STMN2 | 5.507550779 | 0.003911394 | 0.020219659 |
| ENSG00000093134 | VNN3 | 4.385031643 | 0.003932935 | 0.020312337 |
| ENSG00000075673 | ATP12A | -3.717752898 | 0.003939349 | 0.02034079 |
| ENSG00000250748 | AC025419.1 | -5.503756738 | 0.003944403 | 0.020362211 |
| ENSG00000245869 | AP004609.1 | -3.290717363 | 0.003985991 | 0.020553309 |
| ENSG00000259760 | AC015660.2 | 3.657345964 | 0.003997078 | 0.020591593 |
| ENSG00000138823 | MTTP | -4.690737549 | 0.004045287 | 0.020792314 |
| ENSG00000236747 | LINC01282 | 5.782952045 | 0.004049913 | 0.020811331 |
| ENSG00000280429 | AF001548.3 | -3.047026292 | 0.004089766 | 0.020992142 |
| ENSG00000225885 | AC023590.1 | 3.678544997 | 0.004176826 | 0.021360991 |
| ENSG00000249978 | TRGV7 | 3.556479074 | 0.004185356 | 0.021390021 |
| ENSG00000234638 | AC053503.4 | -5.455476647 | 0.004256603 | 0.021685146 |
| ENSG00000279430 | AL590560.2 | -5.544625707 | 0.004284503 | 0.02179765 |
| ENSG00000273355 | AP000894.4 | -5.401810402 | 0.004301743 | 0.021880408 |
| ENSG00000171094 | ALK | 5.322620198 | 0.004303368 | 0.021883725 |
| ENSG00000222033 | LINC01124 | -4.158101272 | 0.004327773 | 0.02197801 |
| ENSG00000243069 | ARHGEF26-AS1 | -3.190333473 | 0.004492205 | 0.022700273 |
| ENSG00000115616 | SLC9A2 | -3.864749752 | 0.004497207 | 0.022720442 |
| ENSG00000254847 | AC009806.1 | -5.554837845 | 0.004505571 | 0.022752477 |
| ENSG00000182674 | KCNB2 | -3.068194935 | 0.004551882 | 0.022939974 |
| ENSG00000100448 | CTSG | -3.020996537 | 0.004580683 | 0.023064449 |
| ENSG00000261786 | AC006058.1 | -3.455601902 | 0.004633005 | 0.023280981 |
| ENSG00000214867 | SRSF9P1 | 3.19224174 | 0.004640633 | 0.023308049 |
| ENSG00000117215 | PLA2G2D | 6.869522543 | 0.004651384 | 0.023339809 |
| ENSG00000221887 | HMSD | -5.404780011 | 0.00467708 | 0.023434378 |
| ENSG00000224034 | LINC02561 | 3.966994621 | 0.004682433 | 0.02344553 |
| ENSG00000211697 | TRGV5 | 3.761767712 | 0.004724103 | 0.023622615 |
| ENSG00000171517 | LPAR3 | 3.696672825 | 0.004748915 | 0.023741406 |
| ENSG00000226530 | AL158055.1 | -3.783707732 | 0.004795087 | 0.023940304 |
| ENSG00000165887 | ANKRD2 | -3.047242079 | 0.004863447 | 0.02421708 |
| ENSG00000267344 | AC003070.1 | 3.015753153 | 0.004876677 | 0.024252625 |
| ENSG00000173404 | INSM1 | 6.462729801 | 0.004878135 | 0.024252625 |
| ENSG00000211892 | IGHG4 | 5.518973286 | 0.004914911 | 0.024403092 |
| ENSG00000187783 | TMEM72 | 5.698507557 | 0.004957141 | 0.024591051 |
| ENSG00000138115 | CYP2C8 | -5.305397231 | 0.005114375 | 0.025197219 |
| ENSG00000237115 | AL139805.1 | -3.747847495 | 0.005120714 | 0.025213376 |
| ENSG00000170128 | GPR25 | 4.989912386 | 0.005127741 | 0.025242451 |
| ENSG00000165973 | NELL1 | 5.139765661 | 0.005145932 | 0.025315373 |
| ENSG00000153930 | ANKFN1 | -5.491526196 | 0.005159516 | 0.025360436 |
| ENSG00000226435 | ANKRD18DP | 4.367076295 | 0.005172877 | 0.025414568 |
| ENSG00000066294 | CD84 | 3.684030732 | 0.005246064 | 0.025725561 |
| ENSG00000230707 | AL589987.1 | -4.6125497 | 0.005306324 | 0.02597373 |
| ENSG00000067842 | ATP2B3 | -3.161360833 | 0.005342996 | 0.026124797 |
| ENSG00000166183 | ASPG | -5.902877014 | 0.005391397 | 0.026332831 |
| ENSG00000251191 | LINC00589 | 5.687498198 | 0.005461363 | 0.026634066 |
| ENSG00000211637 | IGLV4-69 | 3.020505246 | 0.005547655 | 0.026963963 |
| ENSG00000259446 | AC055874.1 | -3.942849897 | 0.005569615 | 0.027050525 |
| ENSG00000260230 | FRRS1L | -4.855811792 | 0.005623286 | 0.02727436 |
| ENSG00000257221 | AC007569.1 | 5.669254902 | 0.005690519 | 0.027542554 |
| ENSG00000226281 | AL031123.1 | 5.141385197 | 0.005718121 | 0.027646396 |
| ENSG00000224661 | AC010907.1 | -5.368564879 | 0.005750972 | 0.027781336 |
| ENSG00000231367 | AC011247.1 | -3.483938892 | 0.005800335 | 0.027989726 |
| ENSG00000231852 | CYP21A2 | -5.262146968 | 0.005877985 | 0.028281586 |
| ENSG00000169218 | RSPO1 | -4.907659314 | 0.005890056 | 0.028319367 |
| ENSG00000134028 | ADAMDEC1 | 4.616391441 | 0.005917843 | 0.028404385 |
| ENSG00000266340 | AC138207.7 | 4.965751788 | 0.005970042 | 0.028612349 |
| ENSG00000272079 | AC004233.3 | 4.919679552 | 0.006075544 | 0.02904353 |
| ENSG00000198515 | CNGA1 | -5.918306764 | 0.006085494 | 0.02906448 |
| ENSG00000179750 | APOBEC3B | 3.477863238 | 0.006098476 | 0.029097482 |
| ENSG00000198203 | SULT1C2 | 4.291991037 | 0.006272308 | 0.029768235 |
| ENSG00000223865 | HLA-DPB1 | 3.602920687 | 0.006277422 | 0.029780674 |
| ENSG00000187546 | AGMO | -3.936309463 | 0.006393352 | 0.030234977 |
| ENSG00000184144 | CNTN2 | -4.769828156 | 0.006413353 | 0.030310437 |
| ENSG00000180383 | DEFB124 | -4.532552916 | 0.006448838 | 0.030458941 |
| ENSG00000012124 | CD22 | 4.08420534 | 0.006471241 | 0.030547307 |
| ENSG00000169085 | VXN | -4.561603677 | 0.006474132 | 0.030552742 |
| ENSG00000279811 | AC093330.2 | 3.063333064 | 0.006606466 | 0.031063172 |
| ENSG00000260776 | AC104758.3 | -4.739037405 | 0.00660992 | 0.031063172 |
| ENSG00000266256 | LINC00683 | -4.655732691 | 0.006713082 | 0.031495323 |
| ENSG00000237541 | HLA-DQA2 | 3.668096565 | 0.006758296 | 0.031667806 |
| ENSG00000283752 | AC241585.3 | 5.639274033 | 0.006775665 | 0.031729359 |
| ENSG00000197360 | ZNF98 | -4.900354492 | 0.006794925 | 0.031799685 |
| ENSG00000120457 | KCNJ5 | 3.995075787 | 0.006816188 | 0.031872658 |
| ENSG00000168333 | PPDPFL | -4.547425749 | 0.006857574 | 0.032032878 |
| ENSG00000137561 | TTPA | -5.187454327 | 0.0069246 | 0.032317704 |
| ENSG00000108342 | CSF3 | -3.992088621 | 0.006971318 | 0.032496689 |
| ENSG00000285108 | AC103718.1 | 5.447281076 | 0.006977154 | 0.032517151 |
| ENSG00000234754 | C1orf140 | -5.276882839 | 0.006996388 | 0.032600035 |
| ENSG00000229436 | AC073850.1 | 3.925292734 | 0.007013844 | 0.032674601 |
| ENSG00000248964 | AC131254.1 | 4.510409243 | 0.007050427 | 0.032804254 |
| ENSG00000279036 | AC015656.1 | -5.184235111 | 0.007053556 | 0.032805237 |
| ENSG00000155816 | FMN2 | -4.873347859 | 0.007087107 | 0.032934033 |
| ENSG00000189068 | VSTM1 | 5.597195748 | 0.007133606 | 0.033122733 |
| ENSG00000238042 | LINC02257 | 5.597195748 | 0.007133606 | 0.033122733 |
| ENSG00000211611 | IGKV6-21 | 5.597195748 | 0.007133606 | 0.033122733 |
| ENSG00000130173 | ANGPTL8 | -5.318360714 | 0.007154863 | 0.033194017 |
| ENSG00000197721 | CR1L | 4.883277681 | 0.007169484 | 0.033234422 |
| ENSG00000127377 | CRYGN | -3.117884166 | 0.00717742 | 0.033262613 |
| ENSG00000229558 | SACS-AS1 | -3.569110687 | 0.007178524 | 0.033262613 |
| ENSG00000215808 | LINC01139 | -4.098092506 | 0.007282503 | 0.033695811 |
| ENSG00000169894 | MUC3A | 3.420579229 | 0.007346193 | 0.033955567 |
| ENSG00000240671 | IGKV1-8 | 5.943407558 | 0.00736295 | 0.03401205 |
| ENSG00000277215 | SPANXA2-OT1 | -4.852164579 | 0.007421779 | 0.034227554 |
| ENSG00000187398 | LUZP2 | -4.051608885 | 0.00746495 | 0.034384338 |
| ENSG00000275585 | AC241377.2 | 3.023322907 | 0.007518186 | 0.03458748 |
| ENSG00000156886 | ITGAD | 5.438089385 | 0.007523627 | 0.034604991 |
| ENSG00000089169 | RPH3A | 4.233487582 | 0.007540211 | 0.034659999 |
| ENSG00000237233 | TMEM26-AS1 | 4.959524607 | 0.007584798 | 0.034815137 |
| ENSG00000090382 | LYZ | 4.197009804 | 0.007588666 | 0.034825783 |
| ENSG00000168702 | LRP1B | -5.653817044 | 0.007603414 | 0.034860353 |
| ENSG00000274395 | AC126177.8 | 4.974884536 | 0.007654778 | 0.035071922 |
| ENSG00000226145 | KRT16P6 | -3.506904062 | 0.007687519 | 0.035186087 |
| ENSG00000263513 | FAM72C | 4.831185861 | 0.007765057 | 0.035475993 |
| ENSG00000211660 | IGLV2-23 | 4.129174206 | 0.007943806 | 0.036138452 |
| ENSG00000223511 | AL683807.1 | 3.030199539 | 0.007982347 | 0.036291759 |
| ENSG00000273259 | AL049839.2 | -3.426937557 | 0.007992306 | 0.036322349 |
| ENSG00000214796 | AC098934.1 | -4.548717531 | 0.008005826 | 0.036369093 |
| ENSG00000102962 | CCL22 | 3.05346142 | 0.008050032 | 0.036533011 |
| ENSG00000211967 | IGHV3-53 | 5.553666207 | 0.008062883 | 0.036576566 |
| ENSG00000224809 | BEND3P2 | 5.279029817 | 0.008149931 | 0.036852504 |
| ENSG00000026751 | SLAMF7 | 3.334775858 | 0.00815796 | 0.036881393 |
| ENSG00000275476 | AC009318.4 | -5.288361448 | 0.008179876 | 0.036965608 |
| ENSG00000258048 | AC073569.2 | -5.124639958 | 0.008230932 | 0.03712918 |
| ENSG00000279940 | AC073862.3 | -5.165419972 | 0.008391996 | 0.037727071 |
| ENSG00000279286 | AL133373.2 | -4.485314663 | 0.008502153 | 0.038123213 |
| ENSG00000166407 | LMO1 | -4.540531063 | 0.008599082 | 0.038496426 |
| ENSG00000253852 | AC011383.1 | -5.15509747 | 0.008607677 | 0.038519571 |
| ENSG00000254290 | AC124067.4 | -5.252901988 | 0.008622623 | 0.038571103 |
| ENSG00000186766 | FOXI2 | -5.125864692 | 0.008759777 | 0.039052558 |
| ENSG00000250274 | AC034199.1 | 3.446198214 | 0.008827097 | 0.039267051 |
| ENSG00000179840 | PIK3CD-AS1 | 3.77908066 | 0.008865002 | 0.039388916 |
| ENSG00000271952 | LINC01954 | 3.541330452 | 0.008909083 | 0.039553514 |
| ENSG00000205632 | LINC01310 | -5.166500149 | 0.008945662 | 0.039692402 |
| ENSG00000162951 | LRRTM1 | -5.267505795 | 0.009030855 | 0.039975754 |
| ENSG00000129810 | SGO1 | 3.192752498 | 0.009049258 | 0.040033574 |
| ENSG00000197261 | C6orf141 | -3.244944587 | 0.009071762 | 0.040123434 |
| ENSG00000169297 | NR0B1 | -5.325293737 | 0.009077792 | 0.040136122 |
| ENSG00000054803 | CBLN4 | 3.980292691 | 0.009085185 | 0.040153018 |
| ENSG00000272666 | U62317.1 | 3.79926233 | 0.009160738 | 0.040439244 |
| ENSG00000256713 | PGA5 | -5.114476614 | 0.009245659 | 0.040733825 |
| ENSG00000266017 | MIR4477B | 3.414178602 | 0.009480538 | 0.041549185 |
| ENSG00000205846 | CLEC6A | 5.541719852 | 0.009581178 | 0.041916723 |
| ENSG00000234424 | AL353743.4 | 4.764873906 | 0.009602653 | 0.041986166 |
| ENSG00000228168 | HNRNPA1P21 | 4.816094598 | 0.009650405 | 0.042145789 |
| ENSG00000165694 | FRMD7 | -4.361092472 | 0.009681443 | 0.042264922 |
| ENSG00000211734 | TRBV5-1 | 3.37616736 | 0.009692587 | 0.042297149 |
| ENSG00000182463 | TSHZ2 | -4.84112645 | 0.009698516 | 0.0423066 |
| ENSG00000160183 | TMPRSS3 | 4.079044312 | 0.00971975 | 0.042358141 |
| ENSG00000187037 | GPR141 | 3.028576886 | 0.009757149 | 0.042494052 |
| ENSG00000237515 | SHISA9 | -5.098250317 | 0.009930417 | 0.04313454 |
| ENSG00000246022 | ALDH1L1-AS2 | -3.198482136 | 0.010248168 | 0.044402803 |
| ENSG00000205057 | CLLU1OS | 5.606094774 | 0.010270297 | 0.044490111 |
| ENSG00000211771 | TRBJ2-7 | 4.736956101 | 0.010401571 | 0.044946221 |
| ENSG00000165509 | MAGEC3 | -3.051559386 | 0.010426288 | 0.045018421 |
| ENSG00000100362 | PVALB | 3.649898109 | 0.010476392 | 0.045214406 |
| ENSG00000131668 | BARX1 | 4.813618013 | 0.010600494 | 0.045682887 |
| ENSG00000177519 | RPRM | -5.328943744 | 0.010618479 | 0.045746791 |
| ENSG00000006128 | TAC1 | -5.154331991 | 0.010782874 | 0.046291424 |
| ENSG00000184471 | C1QTNF8 | -6.023476697 | 0.01079286 | 0.046307732 |
| ENSG00000218809 | AL391903.1 | 3.224788352 | 0.010967177 | 0.046867904 |
| ENSG00000140057 | AK7 | -3.205475164 | 0.011068799 | 0.047266251 |
| ENSG00000160339 | FCN2 | -5.349409739 | 0.011077575 | 0.047294747 |
| ENSG00000224041 | IGKV3D-15 | 3.670075269 | 0.011109114 | 0.047411396 |
| ENSG00000270606 | PPIAP52 | -4.591377875 | 0.011192397 | 0.047739649 |
| ENSG00000138152 | BTBD16 | -4.409557809 | 0.011232419 | 0.047892189 |
| ENSG00000261161 | AC009154.1 | -3.096510807 | 0.011242672 | 0.047917733 |
| ENSG00000224361 | AC011239.1 | -5.099463109 | 0.011320456 | 0.048157981 |
| ENSG00000228140 | AL031283.1 | 3.272480579 | 0.011458418 | 0.048623695 |
| ENSG00000274128 | AC027807.2 | -3.863355656 | 0.011460203 | 0.048623695 |
| ENSG00000123500 | COL10A1 | 3.306808552 | 0.011481243 | 0.048703774 |
| ENSG00000257890 | AC011595.2 | -5.2304036 | 0.011485527 | 0.048712758 |
| ENSG00000260944 | FOXC2-AS1 | -4.407071416 | 0.011560322 | 0.049011492 |
| ENSG00000144130 | NT5DC4 | 3.489784502 | 0.01172738 | 0.049626194 |
